# Examining intergenerational risk factors for conduct problems using polygenic scores in the Norwegian Mother, Father and Child Cohort Study

**DOI:** 10.1038/s41380-023-02383-7

**Published:** 2024-01-16

**Authors:** Leonard Frach, Wikus Barkhuizen, Andrea G. Allegrini, Helga Ask, Laurie J. Hannigan, Elizabeth C. Corfield, Ole A. Andreassen, Frank Dudbridge, Eivind Ystrom, Alexandra Havdahl, Jean-Baptiste Pingault

**Affiliations:** 1 Department of Clinical, Educational & Health Psychology, Division of Psychology & Language Sciences, Faculty of Brain Sciences, University College London, London, UK; 2 Social, Genetic and Developmental Psychiatry Centre, Institute of Psychiatry, Psychology & Neuroscience, King’s College London, London, UK; 3 Center for Genetic Epidemiology and Mental Health, Norwegian Institute of Public Health, Oslo, Norway; 4 PROMENTA Research Center, Department of Psychology, University of Oslo, Oslo, Norway; 5 Nic Waals Institute, Lovisenberg Diaconal Hospital, Oslo, Norway; 6 Population Health Sciences, Bristol Medical School, University of Bristol, Bristol, UK; 7 NORMENT Centre, Division of Mental Health and Addiction, Oslo University Hospital & Institute of Clinical Medicine, University of Oslo, Oslo, Norway; 8 KG Jebsen Centre for Neurodevelopmental disorders, University of Oslo and Oslo University Hospital, Oslo, Norway; 9 Department of Population Health Sciences, University of Leicester, Leicester, UK; 10 NIHR Leicester Biomedical Research Centre, University of Leicester, Leicester, UK

**Keywords:** Psychiatric disorders, Psychology, Genetics

## Abstract

The aetiology of conduct problems involves a combination of genetic and environmental factors, many of which are inherently linked to parental characteristics given parents’ central role in children’s lives across development. It is important to disentangle to what extent links between parental heritable characteristics and children’s behaviour are due to transmission of genetic risk or due to parental indirect genetic influences via the environment (i.e., *genetic nurture*). We used 31,290 genotyped mother-father-child trios from the Norwegian Mother, Father and Child Cohort Study (MoBa), testing genetic transmission and genetic nurture effects on conduct problems using 13 polygenic scores (PGS) spanning psychiatric conditions, substance use, education-related factors, and other risk factors. Maternal or self-reports of conduct problems at ages 8 and 14 years were available for up to 15,477 children. We found significant genetic transmission effects on conduct problems for 12 out of 13 PGS at age 8 years (strongest association: PGS for smoking, β = 0.07, 95% confidence interval = [0.05, 0.08]) and for 4 out of 13 PGS at age 14 years (strongest association: PGS for externalising problems, β = 0.08, 95% confidence interval = [0.05, 0.11]). Conversely, we did not find genetic nurture effects for conduct problems using our selection of PGS. Our findings provide evidence for genetic transmission in the association between parental characteristics and child conduct problems. Our results may also indicate that genetic nurture via traits indexed by our polygenic scores is of limited aetiological importance for conduct problems—though effects of small magnitude or effects via parental traits not captured by the included PGS remain a possibility.

## Introduction

Conduct problems include a variety of rule and norm breaking behaviours such as theft or aggressive behaviour and can develop during childhood and adolescence. Severe conduct problems can be diagnosed as conduct disorder, which has a worldwide prevalence of 2–3% [1, 2]. Furthermore, individuals with conduct problems in childhood can continue to experience conduct problems, mental health problems and a range of adverse outcomes during adolescence and beyond [3], highlighting the importance of early and effective prevention or intervention strategies.

**Fig. 1:**
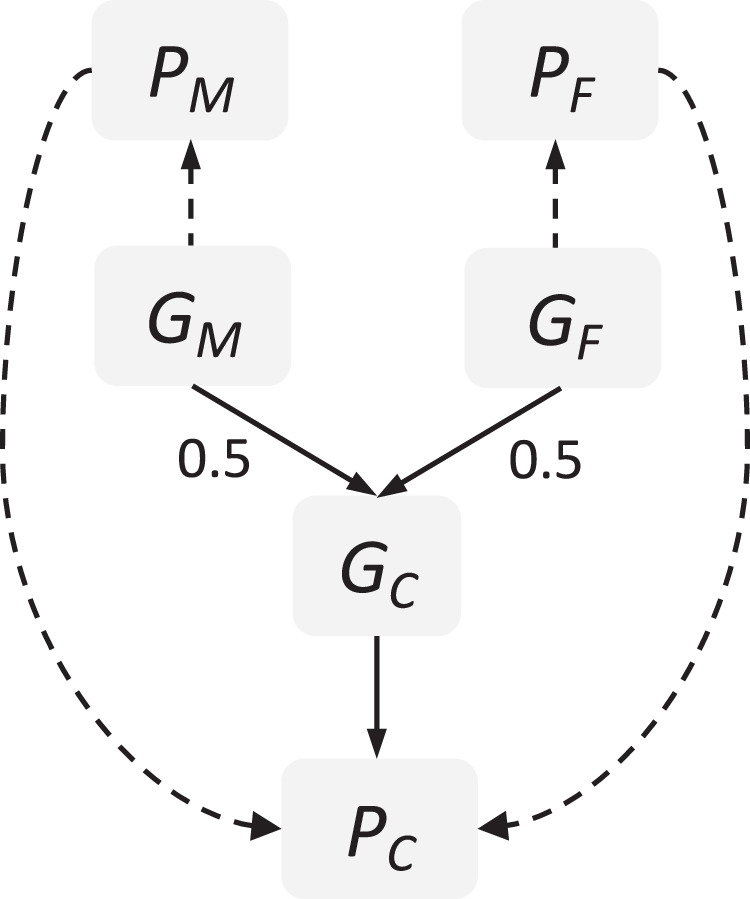
Associations Between Parental Genotypes and Offspring Phenotype. If associations between parental genotypes (*G*_*M*_ or *G*_*F*_) and child phenotype (*P*_*C*_) persist after controlling for child genotype (*G*_*C*_), this could indicate genetic nurture effects working through parental phenotype (*P*_*M*_ or *P*_*F*_). For mothers, genetic nurture effects reflect the pathway *G*_*M*_ → *P*_*M*_ → *P*_*C*_, while genetic transmission is *G*_*M*_ → *G*_*C*_ → *P*_*C*_. The child direct genetic effect is *G*_*C*_ → *P*_*C*_. We note that such ‘indirect genetic effects’ from parental genotype may not purely reflect genetic nurture effects, but can also arise for example, from population stratification. However, ‘indirect genetic effects’ could be a confusing term here, as genetic transmission effects are also indirect from a statistical viewpoint (effects of parental polygenic score are mediated via child polygenic scores), which is why we chose the terminology of ‘genetic nurture effects’ here. *C* = child, *F* = father, *G* = genotype, *M* = mother, *P* = phenotype.

Several individual and parental risk factors for conduct problems have been suggested, which may differ between childhood and adolescence. While some individual risk factors are associated with conduct problems already in childhood, e.g., low cognitive functioning, impulsiveness and hyperactivity [4–6], other factors have been suggested as risk factors in adolescence, e.g., peer deviance, substance use and psychiatric traits like depression [5, 7]. Furthermore, parental risk factors are typically classified as environmental risk factors for the offspring. For example, harsh parenting and substance use during pregnancy are associated with conduct problems in childhood [7–10], whereas parental substance abuse is associated with conduct problems in adolescence [11]. Factors associated with conduct problems in both childhood and adolescence include childhood maltreatment, early motherhood, low parental income and social disadvantage and parental psychiatric conditions (e.g., maternal depression or anxiety), among others [7, 10, 12–16]. Associations between such parental risk factors and conduct problems might reflect causal processes (i.e., intervening on the risk factor would result in a change in conduct problems). However, it is challenging to establish whether associations between parental risk factors and conduct problems reflect a genuine causal effect of the parental risk factor via an environmental route of transmission (e.g., maternal depression can affect parenting, which, in turn, influences conduct problems). This is because genetic factors also contribute to conduct problems, with a heritability estimated to be ~50% from twin studies on broad conduct disorder symptoms [17, 18]. As parental risk factors are also heritable (e.g., heritability of ~37% for depression [19]) this raises the possibility that intergenerational associations partly reflect genetic transmission (effects through transmission of genetic variants from parents to their child), as well as causal effects or other sources of confounding. Genetic information can also be used to detect an environmental route of transmission. Associations between parental genetic factors and child outcomes can arise from *genetic nurture* effects, i.e., indirect genetic effects from non-transmitted parental genes on their child’s outcomes (see Fig. 1). These genetic nurture effects are independent of genetic transmission and thus reflect an environmental route of transmission.

Genetic transmission and genetic nurture effects can be disaggregated for example, using methods capitalising on measured genetics of mother-father-child trios [20–25], adoption and sibling designs [26–29]. For example, trio-GCTA [22] uses measured genetics of mother-father-child trios (henceforth called trios) to decompose the variance of a phenotype into overall contributions of direct genetic and (indirect) genetic nurture effects. While the discovery of overall genetic nurture effects is of interest, it does not pinpoint the specific parental characteristics underlying these observed genetic nurture effects. There has been a growing interest in the use of polygenic scores in within-family designs [20, 21, 23, 29–31]. Polygenic scores are weighted sum scores of an individual’s alleles associated with a disorder or trait; they are often used in downstream analysis of genome-wide association studies (GWAS), for example, for prediction or to identify potential mechanisms underlying genetic associations [32]. Modelling polygenic scores of children and their parents allows us to distinguish genetic nurture effects from genetic transmission [33]. Polygenic scores can be indicative of parental characteristics that may causally influence their offspring’s behaviour (e.g., the maternal polygenic score for depression is associated with maternal depression, which, in turn, can have an environmental influence on the child’s behaviour). As discussed in previous research [34], polygenic scores can be used as genetic proxies for specific parental traits without directly including parental phenotypes. From the viewpoint of intergenerational psychiatry and developmental psychopathology research, identifying specific parental factors that drive genetic nurture effects is essential to better understand the intergenerational transmission of risk for conduct problems. In addition, it can help characterising potential intervention targets to improve offspring’s outcomes.

Recent studies used polygenic scores to study genetic nurture effects for ADHD [35, 36], substance use [37, 38], aggression [39] and educational outcomes [40]. Both trio-GCTA and polygenic scores have been used to study genetic transmission and genetic nurture effects for conduct problems or related phenotypes such as externalising behaviours. One Dutch study used polygenic scores for externalising problems in trios to predict externalising behaviours in childhood and young adulthood, but did not find significant genetic nurture effects [41]. In contrast, another study using the polygenic score for externalising problems found some evidence for genetic nurture effects on externalising problems in adolescents in a smaller subsample of individuals of European ancestry [42]. Furthermore, indirect genetic effects of parents on children’s conduct problems at age 8 years were reported using trio-GCTA (about 7% variance explained for each parent) in addition to genetic transmission effects [43], which suggests some contribution of genetic nurture for conduct problems. Several factors can explain such discrepancies in previous research. Different methods have been used, i.e., identifying general genetic nurture effects with trio-GCTA versus more specific genetic nurture effects using a single polygenic score for externalising problems. Importantly, sample size may also explain the apparent discrepancies (600–800 trios for polygenic score studies, 9700 for trio-GCTA study). Previous studies were underpowered to detect modest genetic nurture effects or may have found false-positive effects. Furthermore, the developmental stage (childhood, adolescence or young adulthood) as well as sample characteristics like phenotype definition may explain the diverging results.

In this study we use genotyped trios of the Norwegian Mother, Father and Child Cohort Study (MoBa) [44, 45]. Adding to the existing literature, we investigate the relative importance of genetic transmission and genetic nurture effects for conduct problems in one of the largest cohorts of genotyped trios. Our large sample has sufficient power despite multiple testing correction. This allows us to examine multiple polygenic scores for 13 traits and disorders, which correspond to parental risk factors that have been consistently reported in quasi-experimental research on conduct problems [10] (increasing the possibility of a true causal effect of these parental factors). Furthermore, we investigate associations in childhood (age 8 years) and adolescence (age 14 years), adding to the literature on genetic nurture and genetic transmission in the earlier developmental stages of conduct problems and externalising behaviours. Effects might differ across developmental stages, as externalising behaviours such as conduct problems likely express differently in childhood and adolescence [46] and genetic effects on externalising behaviours may differ across development [47].

In addition to our main analyses, *direct genetic effects* of children’s own polygenic scores on their outcome are obtained when adjusting for parental genotypes. Compared to population estimates from studies of unrelated individuals, direct genetic effects can be more easily interpreted because they only reflect the genetic effects originating in the child after accounting for influences such as genetic nurture, assortative mating or residual population stratification [48, 49]. This is important because although we use the term genetic nurture effects to describe indirect genetic effects, these could also partly reflect assortative mating or residual population stratification. The estimation of direct genetic effects on conduct problems using multiple polygenic scores further adds to the literature, as comparisons between a range of within-family polygenic score estimates and unadjusted (population) estimates have not been carried out for conduct problems.

## Materials and methods

### Sample description

MoBa [44, 45] is a population-based pregnancy cohort study conducted by the Norwegian Institute of Public Health. Participants were recruited from all over Norway from 1999 to 2008. The women consented to participation in 41% of the pregnancies. The cohort includes approximately 114,500 children, 95,200 mothers and 75,200 fathers. Blood samples were obtained from both parents during pregnancy and from mothers and children (umbilical cord) at birth [50]. The current study is based on version 12 of the quality-assured data files released for research in January 2019. The establishment of MoBa and initial data collection was based on a license from the Norwegian Data Protection Agency and approval from The Regional Committees for Medical and Health Research Ethics (REK). The MoBa cohort is now based on regulations related to the Norwegian Health Registry Act. The current study was approved by REK (2016/1702). We further used data from the Medical Birth Registry of Norway (MBRN), a national health registry containing information about all births in Norway, and the Norwegian Patient Registry for multiple imputation of the phenotype data (further described under ‘Multiple imputation of phenotype data’ in the methods and in the supplementary note). The STROBE checklist for reports using cohort studies [51] can be found in Supplementary Table S1.

### Phenotype description

In childhood, we used the eight items from the abbreviated conduct disorder subscale of the Rating Scale for Disruptive Behaviour Disorders (RS-DBD) [52], which were rated from 1 (never/rarely) to 4 (very often) by the children’s mothers at 8 years of age. These eight items were used as indicators for a latent factor of conduct problems, which was used as the outcome in all analyses (see Statistical analyses). In adolescence, self-reports of the RS-DBD conduct disorder subscale were available when participants were 14 years old, and items were rated from 1 (never/rarely in the last year) to 6 (more than 20 times in the last year). Due to the low number of responses for response options 4 (5–10 times), 5 (10–20 times) and 6 on all eight items, we combined response options 4 to 6, which resulted in a 4-point scale comparable to the age 8 time point. In our sample, internal consistency estimates for the conduct disorder subscale in childhood and adolescence were good (α = 0.87 and α = 0.90, respectively, for the ordinal scale).

### Genotyping and quality control of genotype data

Genotyping was performed using the Illumina Global Screening Array, Illumina HumanCoreExome, Illumina HumanOmniExpress, and Illumina InfiniumOmniExpress Arrays. Standard quality control and genotype imputation procedures were applied and described in detail elsewhere [53] and in the supplementary note. The sample was further filtered to only include families with genetic data available for complete trios (i.e., mother, father and child). We removed families with individuals who withdrew consent (updated on 6th June 2023) and closely related individuals across trios based on the proportion of the genome shared identity-by-descent >0.1768 (roughly corresponding to a KING kinship coefficient of 0.0884, which differentiates between first and second degree relatives), resulting in a final sample of 31,290 genotyped trios.

### Selection of GWAS summary statistics

We focused on parental risk factors that have been consistently associated with conduct problems in quasi-experimental studies [7, 10], as these factors are more likely to reflect causal environmental effects that drive potential genetic nurture effects on conduct problems in childhood and adolescence [43]. Furthermore, we chose risk factors for which GWAS existed at the time of analysis that were conducted on individuals of European ancestry, and for which summary statistics were publicly available. GWAS of traits or disorders with a SNP-based heritability (*h*^2^_*SNP*_; variance explained in the outcome by common SNPs, i.e., single nucleotide polymorphisms) of >5% and a *Z*-score above 2 (*h*^2^_*SNP*_ / *SE*_*h*_^2^_*SNP*_) were included as recommended previously [54]. As a result, we included summary statistics from GWAS capturing a variety of parental risk factors for conduct problems in the domains of psychiatric conditions, substance use/abuse, cognitive and education-related factors, or other parental risk factors that could underlie potential genetic nurture effects (Supplementary Table S2). Specifically, we used summary statistics for GWAS on ADHD [55], depression [56], lifetime anxiety disorders (UK biobank) [57], antisocial behaviour (ASB) [58], lifetime smoking [59], problematic alcohol use [60], cannabis use disorder (CUD) [61], cognitive performance [62], educational attainment [62], household income [63], risky behaviours [64] and age at first birth [65]. As sensitivity analysis, we also included a polygenic score derived from a GWAS on broad externalising behaviours [66, 67], which might explain more variance in our conduct problems phenotypes at the cost of being less specific for single parental factors that might be targets for interventions.

Prior to polygenic score analysis, we conducted quality control on the GWAS summary statistics, retaining variants on autosomal chromosomes with a MAF > 0.01 and INFO > 0.8 (where available), and removing ambiguous and duplicated SNPs as recommended [54].

### Polygenic scoring

Polygenic scores for children, mothers and fathers for each trait were computed with LDpred2, a Bayesian method to derive polygenic scores using information on genetic architecture (SNP-based heritability and polygenicity measured as the fraction of causal variants) and on Linkage Disequilibrium (LD) obtained from a reference panel [68]. To compute polygenic scores, recommended quality control steps were followed [69] and, accordingly, variants included were restricted to an extended set of HAPMAP3 variants: https://figshare.com/articles/dataset/LD_reference_for_HapMap3_/21305061. UK Biobank was used as reference LD panel in polygenic score calculations using precomputed LD matrices [69]. Polygenic scores were generated by using the option ‘LDpred2-auto’ and standardised. Finally, polygenic scores were adjusted for population stratification and batch effects by regressing out genotyping batch, plate ID, imputation batch and the first ten principal components of ancestry, as well as sex and year of birth.

### Statistical analyses

All statistical analyses were performed in R [70] v4.1.2 and v3.5.0 (for LDpred2). We used structural equation models (SEM) as implemented in the *lavaan* package v0.6.9 [71] to test the effects of parental and child polygenic scores on a latent factor (conduct problems), indicated by eight ordinal items, using the diagonally weighted least squares estimator with robust standard errors. This allows the control of measurement error and potential issues of using sum scores for non-continuous items [72].

**Fig. 2:**
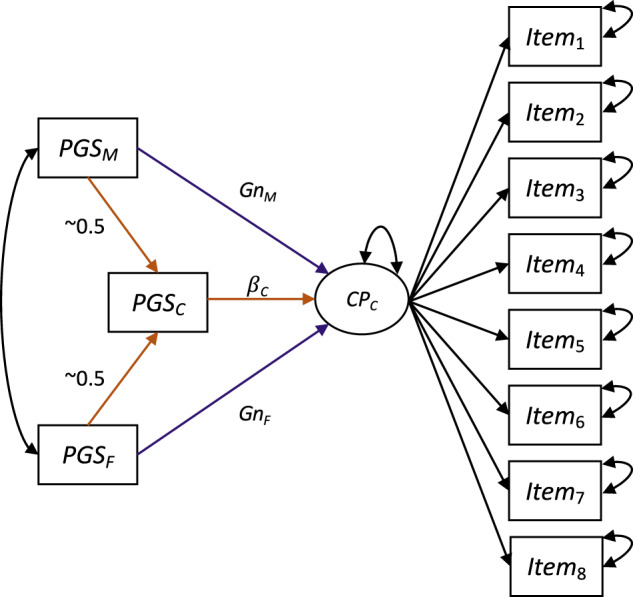
Structural Equation Models. Child conduct problems (*CP*_*C*_) are modelled as a latent factor, indicated by eight ordinal items of the conduct disorder subscale of the RS-DBD. The model is initially analysed separately for the 13 different polygenic scores (e.g., PGS for ADHD, PGS for depression). Genetic transmission effects (orange paths), e.g., for the mothers, are estimated as the pathway *PGS*_*M*_ → *PGS*_*C*_ → *P*_*C*_ which effectively is ~0.5 × β_*C*_ and can be considered as a mediation or indirect effect of parental polygenic scores on child outcomes via the respective child polygenic score. Associations between child and parental polygenic scores vary around the expected value of 0.5, and estimated values are used in the calculation of genetic transmission effects (see Supplementary Table S9). The models allow the maternal and paternal polygenic scores to covary.

We jointly modelled parental and child polygenic scores (trio models), thus dissecting associations with parental polygenic scores into genetic transmission and genetic nurture effects. This was initially done for each trait separately, resulting in 13 models, each model including maternal, paternal and child polygenic scores for that trait. As sensitivity analysis, we also estimated a multi polygenic score model including all 36 polygenic scores for the main analysis using complete data at age 8 years (12 polygenic scores per family member, not including the polygenic scores for externalising problems, which can be conceived as a combination of other polygenic scores included). Genetic transmission for each risk factor was estimated as the effect of parental polygenic scores on child conduct problems via the respective child polygenic score, which is effectively ~0.5 × β_*PGSchild*_, where β_*PGSchild*_ is the direct genetic effect of the child’s own polygenic score. Genetic nurture effects were estimated as the residual association between the parental polygenic score and child conduct problems after adjusting for the respective child polygenic score (see Fig. 2). For comparison, we estimated unadjusted models (one polygenic score per model for each family member separately, i.e., 39 models) without controlling for the polygenic scores of the other family members. We note that estimates from unadjusted models have also been referred to as population estimates that are typically examined in studies of unrelated individuals [73]. Summing the genetic transmission effect and the genetic nurture effect (trio models) should equal the observed association between a parental polygenic score and the child outcome without adjusting for the child polygenic score (unadjusted models). In addition, if unadjusted models including only the child polygenic score differ from the direct genetic effects obtained from the trio models, this indicates the presence of indirect genetic effects such as genetic nurture. *P*-values for each type of effects tested were adjusted by applying the false-discovery rate (FDR) correction [74], i.e., *N*_*traits*_ × *N*_*effects*_. *P*-values for genetic transmission estimates were adjusted for 13 tests and genetic nurture effects were adjusted for 26 tests (13 PGS and one effect per parent). Associations with *q*-values (FDR corrected *p*-values) <0.05 were considered significant. In addition, we performed power calculations for our polygenic score analyses (similar to ref. [75]), which can be found in the supplementary materials.

### Sensitivity analysis using multiple imputation of phenotype data

Out of 31,290 trios with genetic data, 15,477 families also had relevant phenotype data at age 8 years, i.e., about 50% missing. As data was likely not missing completely at random due to previously reported selective attrition [76], we imputed missing data at age 8 years by multiple imputation using chained equations as implemented in the R package *mice* v3.13.0 [77], and followed recent guidance [78], creating 100 imputed data sets with 30 iterations to achieve convergence, and pooling results from the imputed data sets. Age 14 year data were not imputed due to large degrees of missingness (8035 out of 31,290 trios with genetic data also had relevant phenotype data at age 14 years). Details on the imputation process, including additional variables used for imputation and results using imputed data can be found in the supplementary note and in Supplementary Tables S3 and S6. Convergence plots and distributions of observed and imputed data are shown in Supplementary Figs. S1 and S2.

## Results

The genotyped sample consisted of 31,290 children (49.2% female) and their parents. Phenotype data at age 8 years for at least one item for conduct problems was available for a subset of 15,477 children (50.5% missingness), of which 15,320 children had available data on all eight items of the RS-DBD conduct disorder subscale. At age 14 years, data were available on all eight items for 7883 individuals (53.5% female; see Table 1).

**Table 1:** Descriptive statistics of the full sample and subsample with complete phenotype data.

Variable		*N* (% missing)	Mean (*SD*)	Range	*N* (% missing)	Mean (*SD*)^a^	Range
Genetic data (trios)		31,290					
Conduct disorder subscale		Age 8			Age 14		
	Any item	15,477 (50.5)			8035 (74.3)		
	All items	15,320 (51.0)			7883 (74.8)		
Item 1	*Bullies/threatens/intimidates*	15,435 (50.6)	1.20 (0.47)	1–4	8019 (74.4)	1.13 (0.53)	1–6
Item 2	*Initiates physical fights*	15,437 (50.7)	1.11 (0.36)	1–4	8020 (74.4)	1.05 (0.31)	1–6
Item 3	*Physically cruel*	15,427 (50.6)	1.27 (0.53)	1–4	8010 (74.4)	1.12 (0.49)	1–6
Item 4	*Injured animals*	15,452 (50.6)	1.02 (0.17)	1–4	8014 (74.4)	1.02 (0.22)	1–6
Item 5	*Stolen items from others*	15,447 (50.6)	1.02 (0.17)	1–3	8010 (74.4)	1.08 (0.44)	1–6
Item 6	*Destroyed property*	15,444 (50.6)	1.05 (0.23)	1–4	8023 (74.4)	1.07 (0.36)	1–6
Item 7	*Truant from school*	15,431 (50.7)	1.01 (0.12)	1–4	8004 (74.4)	1.30 (0.79)	1–6
Item 8	*Used harmful objects*	15,444 (50.6)	1.04 (0.22)	1–4	8006 (74.4)	1.02 (0.24)	1–6

	Sample with complete child data 8 y			Genotyped trio sample			Full MoBa sample^b^		
	*N*	Mean (*SD*)	Range	*N*	Mean (*SD*)	Range	*N*	Mean (*SD*)	Range
Maternal age (baseline)	15,320	30.53 (4.31)	17–45	31,290	30.19 (4.49)	17–45	111,635	30.13 (4.65)	17–45
Paternal age (baseline)	15,320	32.84 (5.08)	18–59	31,288	32.60 (5.22)	18–59	111,121	32.73 (5.47)	18–59

	Mothers	Fathers	Mothers	Fathers	Mothers	Fathers
Parental education (baseline)	*N* (%)	*N* (%)	*N* (%)	*N* (%)	*N* (%)	*N* (%)
Lower secondary education	172 (1.2)	400 (2.8)	599 (2.1)	963 (3.5)	2749 (2.8)	2969 (4.0)
Upper secondary school, basic	426 (2.9)	725 (5.1)	1230 (4.3)	1600 (5.8)	4946 (5.1)	4604 (6.2)
Vocational upper secondary school	1533 (10.6)	3370 (23.7)	3626 (12.8)	7143 (26.1)	12,569 (13.0)	19,166 (25.9)
Upper secondary school, completed	1798 (12.4)	1708 (12.0)	4013 (14.1)	3432 (12.5)	14,283 (14.8)	9160 (12.4)
Higher education, undergraduate level	6634 (45.8)	4186 (29.5)	12,060 (42.4)	7651 (27.9)	39,405 (40.8)	19,967 (27.0)
Higher education, graduate level	3925 (27.1)	3818 (26.9)	6891 (24.2)	6620 (24.2)	22,567 (23.4)	18,087 (24.5)

^a^Mean and SD calculated based on the truncated items (responses options 1–4).

^b^Full sample here refers to families with unique pregnancy IDs.

SNP-based heritability estimates, standard errors as well as the resulting *Z*-scores from the GWAS summary statistics are shown in Supplementary Table S2 and bivariate correlations between the 13 child polygenic scores are shown in Supplementary Table S4.

**Fig. 3:**
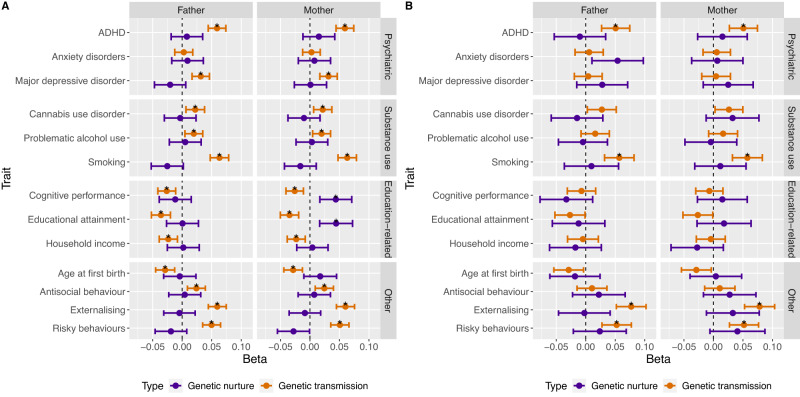
Main Results Using Complete Data at Ages 8 (A) and 14 Years (B). Standardised regression coefficients obtained from the trio models as well as 95% confidence intervals are shown on the x-axis. Asterisks indicate associations with *q* < 0.05.

**Fig. 4:**
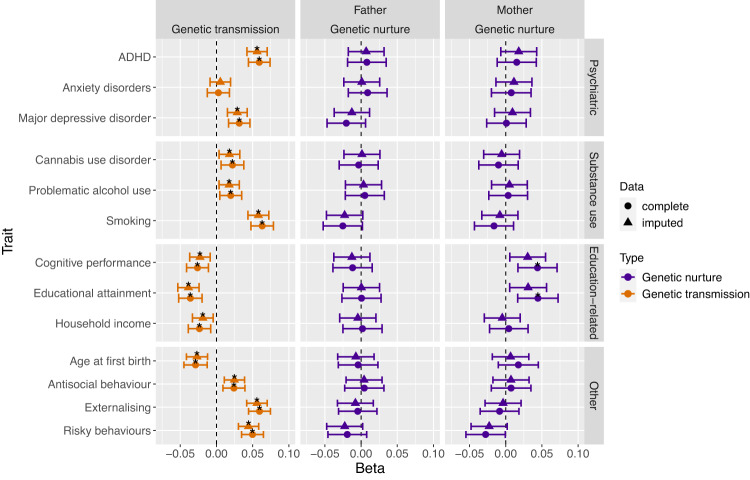
Comparison of Results Using Complete vs Imputed Data at Age 8 Years. Standardised regression coefficients obtained from the trio models with complete data vs imputed data, as well as 95% confidence intervals are shown on the x-axis. Asterisks indicate associations with *q* < 0.05. We only show the genetic transmission effect estimates for one parent, as they are almost identical with no visual differences (see Supplementary Tables S5 and S6).

Using complete data at age 8 years, our measurement model of the latent factor for conduct problems showed good fit, measured by the comparative fit index (CFI), Tucker-Lewis index (TLI), the standardised mean square residual (SMSR) and the root mean square error of approximation (RMSEA; CFI = 0.99, TLI = 0.99, SMSR = 0.08 and RMSEA = 0.03). At age 14 years, the measurement model also showed good model fit (RMSEA; CFI = 0.99, TLI = 0.99, SMSR = 0.05 and RMSEA = 0.02). Main results using complete data, including the standardised regression coefficients obtained from SEMs for each polygenic score are shown in Fig. 3a and summarised in Supplementary Table S5. Results using imputed data can be found in the supplementary note, Fig. 4 and Supplementary Table S6. Results using data at age 14 years can be found in Fig. 3b and in Supplementary Table S7.

### Genetic nurture vs genetic transmission

At age 8 years, we observed significant genetic transmission effects (i.e., effects of parental polygenic score on child conduct problems via the child polygenic score) using all polygenic scores except for anxiety (see Fig. 3 and Supplementary Table S5). As mentioned, these effects were half the size of the direct genetic effects (|β| = 0.02–0.07), given the genetic relatedness between children and their parents—resulting in a correlation of ~0.5 between the child’s polygenic scores and the corresponding polygenic scores of their parents (see Supplementary Table S9 for observed bivariate correlations between parental and child polygenic scores). For both time points, estimates for the direct genetic effects (Supplementary Figs. S3 and S4 and Supplementary Tables S5 and S7) in our models were highly similar to estimates derived from unadjusted models without including the parental polygenic scores (see Supplementary Figs. S5 and S6). At age 14, we also found significant genetic transmission using 4 out of 13 polygenic scores. Genetic effects reflecting the externalising spectrum persisted (e.g., polygenic scores for ADHD, substance use, other externalising behaviours), but we did not find significant genetic transmission using other polygenic scores, for example, for depression or income (Fig. 3b and Supplementary Table S7).

In contrast to genetic transmission effects, estimates for genetic nurture effects were generally much smaller than estimates for genetic transmission, with most point estimates near zero at ages 8 (Fig. 3a and Supplementary Tables S5 and S6) and 14 years (Fig. 3b and Supplementary Table S7). At age 8 years for example, using polygenic scores for ADHD we observed a genetic transmission effect of 0.06, but maternal and paternal genetic nurture effects of 0.008 and 0.015, respectively (i.e., 7–12% of the child direct genetic effect). After multiple testing correction, we found two significant genetic nurture effects on conduct problems—positive associations with the maternal polygenic scores for educational attainment and cognitive performance (β = 0.04, 95% CI [0.02, 0.07] and β = 0.04, 95% CI [0.02, 0.07], respectively). However, these associations were not significant in the corresponding models using the full genotyped sample with imputed phenotype data (see Fig. 4, Supplementary Table S6 and supplementary note). In general however, results using the imputed data sets were highly similar to the results using complete data (*r* = 0.99 for parental effects, *r* > 0.99 for child effects; see Fig. 4 and supplementary materials). At age 14 years, we did not observe any genetic nurture effects (see Fig. 3 and Supplementary Table S7). Estimates from the multi-polygenic score model (all 36 parental and child polygenic scores) showed highly similar results to the main analysis (see Supplementary Table S8).

## Discussion

In this study, we investigated the relative contribution of genetic transmission and genetic nurture effects in the intergenerational risk transmission for conduct problems in childhood and adolescence. We found evidence for genetic transmission, but no strong evidence for genetic nurture effects using a variety of polygenic scores for traits that might drive potential genetic nurture effects for conduct problems.

### Genetic nurture vs genetic transmission

We found evidence for significant genetic transmission in the association between 12 parental polygenic scores and child conduct problems at age 8 years (Supplementary Tables S5 and S6), of which polygenic scores for smoking, ADHD, externalising and for risk-taking behaviour also showed significant associations at age 14 years. Genetic transmission effects on conduct problems using polygenic scores in general can be interpreted as a common underlying genetic aetiology between parental risk factors and child conduct problems. This is important to consider as, for example, genetic effects captured by the polygenic scores for substance use in childhood (age 8 years) are detected in the absence of actual substance use at that age. Such direct effects thus likely reflect broader facets of the externalising spectrum such as impulsivity or self-regulation. These externalising behaviours in turn may have an effect on actual substance use in later developmental stages such as adolescence or young adulthood. In our sensitivity analysis at age 8 years including all 36 polygenic scores (Supplementary Table S8), genetic transmission effects, including for the polygenic scores for substance use, are consistent with our main analyses. For example, the polygenic score for smoking is still associated with child conduct problems after adjusting for all other polygenic scores, such as ADHD or risk behaviours. This confirms the unique genetic contributions of the polygenic scores included in our analyses.

In contrast, our findings do not support genetic nurture as a mechanism underlying associations between these specific parental polygenic scores and children’s conduct problems. In fact, relative effect sizes of genetic nurture estimates (in comparison to child direct genetic effects) were also considerably smaller than reported for other outcomes such as educational attainment, where genetic nurture effects are estimated to be ~40% of the direct genetic effects [40]. However, we did observe positive genetic nurture effects on conduct problems using the maternal polygenic scores for educational attainment and cognitive performance (e.g., higher education polygenic score linked to higher conduct problems). This is in contrast to the genetic transmission effects using the maternal polygenic scores for educational attainment and cognitive performance, which are in the expected direction (negative). However, this finding is not robust across analyses and should thus be considered with caution (further discussions can be found in the supplementary note).

In general, our results suggest that associations between parental polygenic scores for psychiatric conditions, substance use, education-related factors, other risk behaviours, and conduct problems reflect genetic transmission rather than genetic nurture (or other indirect genetic effects due to population stratification or assortative mating [33, 34]). We note that a previous study in a different subsample of MoBa used genetic relatedness matrices and found small genetic nurture effects for conduct problems at age 8 years in addition to genetic transmission [43]. This apparent discrepancy may be resolved by additional replications of both types of studies, potentially indicating one set of false negative or false positive findings, respectively. False negative findings in our approach may stem from a lack of power. Power simulations (supplementary note and Supplementary Figs. S7 and S8) show that we had >0.99 power to detect genetic transmission, and >0.99 power to detect genetic nurture effects which are about 40% of the size of direct genetic effects (based on genetic nurture effect sizes observed for educational outcomes [23, 40]). However, statistical power to detect genetic nurture effects ≤0.03 was below 0.80. The effect sizes and confidence intervals observed in our sample suggest that any true genetic nurture effects on conduct problems should be (substantially) smaller than those observed for educational attainment. It is also possible that both our findings using polygenic scores and previous findings using a variance decoposition approach [43] are true. This would suggest that genetic nurture effects for conduct problems are not captured by our selection of polygenic scores for psychiatric conditions, substance use, education-related factors, and other risk behaviours. In adolescence, we did not find any evidence for genetic nurture effects on conduct problems. This finding is in line with a previous study which did not find genetic nurture effects for externalising behaviours in adolescence [41]. In contrast, another study found evidence for genetic nurture effects on externalising problems in adolescence [42], which may stem from differences in the operationalisation of the externalising phenotypes.

Overall, existing evidence, pending replications, points towards a substantial role of genetic transmission in the intergenerational transmission of risk for conduct problems and, possibly, small environmentally mediated genetic nurture effects. As we did not find convincing evidence for genetic nurture effects via specific parental factors, targeting these parental factors associated with child conduct problems might not be as effective to improve conduct problems of the offspring as could be expected from non-genetically informed studies. This would in turn mean that, for example, instead of intervening on parental factors to improve child conduct problems, intervention efforts should rather be directed towards children by using different modalities, whether through medication, teacher-led or parental involvement where it is practical [79, 80]. For example, such child-directed interventions may focus on the underlying liability to conduct problems. Our results showed that genetic effects related to cannabis use disorder are associated with conduct problems in childhood, which is likely driven by genetic effects on self-regulation or risk-taking behaviour rather than an exclusive effect on the liability to cannabis use disorder. Improving children’s self-regulation and impulsiveness might not only prevent conduct problems, but also severe outcomes in later development, such as cannabis or other substance use disorders.

Our results regarding the relative importance of genetic transmission and genetic nurture effects do not necessarily contradict findings of small environmental effects of parental factors on child conduct problems. Some putative parental risk factors (e.g., low parental education or maternal depression) were consistently associated with conduct problems in quasi-experimental studies [10]. In addition, other genetically informed studies found that environmental factors significantly contribute to the intergenerational transmission of conduct problems [81]. Such parental environmental effects can either be environmentally mediated genetic effects (e.g., genetic nurture effects) or in scenarios where parental risk factors are independent of genetic effects, purely environmental effects. However, as for almost all single risk factors, these effects statistically remain small in magnitude. Using alternative methods like the children-of-twins design [82, 83] and other designs to study intergenerational effects [84] are promising additions to our approach to disentangle genetic and environmental effects of parents on their offspring. Our polygenic score approach does not replace the investigation of parent-child associations using detailed observed parental phenotypes but rather adds to the literature by accounting for genetic transmission in the intergenerational associations between parents and child conduct problems. Future work in large samples on parental (genetic nurture) effects on child conduct problems should include proximal (e.g., parental phenotypic ADHD or depression) and distal mediators (e.g., warm and sensitive parenting) underlying potential genetic nurture effects.

### Direct genetic effects

Direct genetic effects from children’s own genotype on their phenotype are necessary for genetic transmission effects and are part of one pathway from parental polygenic scores to child conduct problems (see Fig. 1). Importantly, associations between child polygenic scores and conduct problems in our study are adjusted for biases arising from genetic nurture effects, population stratification and assortative mating [49] by controlling for parental polygenic scores. This is important, as assortative mating has been reported for the MoBa sample used in our study [85]. In general, all direct genetic effects were of modest size, which is to be expected given previously reported effect sizes of polygenic scores predicting child behavioural and psychological phenotypes, with |β| ≤ 0.16 across both time points [86–89]. At age 8 years, we found direct genetic effects on conduct problems using 12 polygenic scores (Supplementary Fig. S3 and Supplementary Tables S5 and S6). Interestingly, we show that unadjusted estimates are highly similar to adjusted estimates from our trio models (Supplementary Figs. S4 and S5), suggesting that associations between polygenic scores and conduct problems are largely unaffected by genetic nurture, population stratification and assortative mating. This differs from studies focusing on behavioural outcomes such as smoking or education, which found that associations with polygenic scores substantially decrease in magnitude when adjusting for polygenic scores of related individuals [28, 40, 73].

Our results of cross-trait genetic associations are in line with previous research showing that polygenic scores for educational attainment, major depression and other psychiatric traits are associated with other types of childhood psychopathology [90]. Our findings support a common underlying aetiology between conduct problems, psychiatric conditions and other risk factors. While our study is the first to use a within-family design to examine a range of polygenic scores in association with conduct problems, we replicated findings from studies on conduct problems using unrelated individuals. Consistent findings include positive associations between polygenic scores for ADHD, depression, smoking, risky behaviours [86, 87, 91, 92] and conduct problems, and negative associations between polygenic scores for educational attainment, age at first birth and conduct problems, with effect sizes similar to previous studies [88, 89, 93].

The associations between polygenic scores for cannabis use disorder, smoking and for problematic alcohol use, and child conduct problems are in line with the conceptualisation of conduct disorder/problems and substance use disorder as part of an externalising spectrum [94]. This is also consistent with our results of significant associations between the polygenic scores for ADHD, broad antisocial behaviour and for externalising behaviours and conduct problems. Our results converge with recent genomic research on other externalising phenotypes and substance use (disorders), which found substantial genetic overlap across the externalising spectrum [95]. For risk factors beyond the externalising spectrum, positive associations between polygenic scores for depression and child conduct problems support a shared genetic aetiology that may explain the co-occurrence of conduct problems and depression [96]. In addition to phenotypic associations between child conduct problems and socioeconomic factors reported in the literature [3, 10], our results further show that genetic liability to (lower) cognitive performance, education and income itself is associated with conduct problems.

We also found significant direct genetic effects at age 14 years using the polygenic scores for ADHD, smoking, externalising and risk-taking behaviours. For some of the other polygenic scores, results at 14 years showed the same trend as at 8 years but may have failed to reach significance due to a smaller sample size (e.g., for cannabis use disorder or age at first birth). However, estimates for polygenic scores for depression and income, which were significant in the analysis of the 8-year data, were close to zero at age 14 years. This could be interpreted as differential genetic effects on conduct problems across different developmental stages, as has been reported for different externalising phenotypes [47, 97, 98]. For example, new genetic effects could act in adolescence [99, 100] which were potentially not captured by the polygenic scores we used.

In addition, maternal reports were used at age 8 years and adolescents’ self-reports at age 14 years in our study. In line with previous studies measuring conduct problems across time using different raters, conduct problems at ages 8 and 14 years were only moderately correlated (see supplementary note) [92, 100, 101]. As genetic effects on self-reported conduct problems can be smaller compared to parent-reported conduct problems [100, 101], this could also contribute to explain the lower genetic effects at age 14 years in our sample.

Disentangling the observed associations between risk factors and conduct problems into genetic overlap, genetic confounding or causal effects requires follow-up analyses such as colocalization, Mendelian randomisation or genetic sensitivity analysis [33, 102–105]. However, our results suggest that adjustment for indirect genetic effects by including parental polygenic scores might not be necessary to interpret genetic effects on conduct problems.

### Limitations

This study has some limitations. First, studies reported ascertainment bias in MoBa, where self-selection of mothers resulted in an underrepresentation of certain groups of families [106]. Importantly, young mothers and smokers were underrepresented, which both have been reported as parental risk factors for conduct problems. Selection and loss to follow-up in the MoBa sample has been shown to result in both over- and under-estimation of several exposure-outcome associations for ADHD [76], a phenotype closely related to conduct problems. Indeed, we observed that the parental age at birth and the prevalence of highly educated parents were slightly higher in the sample with data on conduct problems than in the whole MoBa sample (Table 1). Multiple imputation of the phenotype data, which is more appropriate than listwise deletion when data is not missing completely at random [107, 108], did not considerably change our results, which suggests that our findings hold robust despite some selective attrition. Importantly, initial participation bias [106] is not accounted for using multiple imputation. Second, our measure of conduct problems at age 8 years was based on maternal reports, which are partly subjective rather than purely objective measures [109]. Third, although we studied a wide range of polygenic scores to index potential parental risk factors that have been empirically and conceptually associated with conduct problems, we could not study all possible risk factors for conduct problems. Specifically, we did not examine any associations with parental behaviours such as maltreatment or harsh parenting, which are well-studied risk factors for conduct problems, because there are no large-scale genome-wide association studies available for these traits from which to derive polygenic scores.

## Conclusion

To conclude, our results suggest that associations between parental polygenic scores and child conduct problems at ages 8 and 14 years predominantly reflect genetic transmission. We cannot rule out the existence of genetic nurture effects for conduct problems; either effects of small magnitude or effects of parental traits not captured by those polygenic scores. Future research in large samples is required to replicate our results as well as results from variance-component approaches for genetic nurture effects on conduct problems. In addition, studies may investigate other powerful polygenic scores to pinpoint potential genetic nurture effects for conduct problems.

### Supplementary information

Supplementary materials

## Data Availability

Data from the Norwegian Mother, Father and Child Cohort Study and the Medical Birth Registry of Norway used in this study are managed by the national health register holders in Norway (Norwegian Institute of public health) and can be made available to researchers, provided approval from the Regional Committees for Medical and Health Research Ethics (REC), compliance with the EU General Data Protection Regulation (GDPR) and approval from the data owners. The consent given by the participants does not open for storage of data on an individual level in repositories or journals. Researchers who want access to data sets for replication should apply through helsedata.no. GWAS summary statistics are publicly available via the Psychiatric Genomics Consortium (https://pgc.unc.edu/), the GWAS Catalog (https://www.ebi.ac.uk/gwas/) and the GWAS Atlas (https://atlas.ctglab.nl/).

## Code availability

The pipeline to compute polygenic scores and the analysis code for the statistical analyses reported here can be found on GitHub (https://github.com/AndreAllegrini/LDpred2) and (https://github.com/LeonardFrach/intergen_conduct).
