# Supplementary material for: Examining intergenerational risk factors for conduct problems using polygenic scores in the Norwegian Mother, Father and Child Cohort Study
Source: Mol Psychiatry. 2024 Jan 16;29(4):951–61. doi: 10.1038/s41380-023-02383-7 (PMC11176059; doi:10.1038/s41380-023-02383-7)
Supplement: Supplementary file 1 — Supplementary materials [file 41380_2023_2383_MOESM1_ESM.pdf]

Supplementary Material for:

Examining Intergenerational Risk Factors for Conduct Problems Using Polygenic Scores in the Norwegian Mother, Father and Child Cohort Study

Leonard Frach[1]*, Wikus Barkhuizen[1], Andrea G. Allegrini[1,2], Helga Ask[3,6], Laurie J. Hannigan[4,5,6], Elizabeth C. Corfield[4,6], Ole A. Andreassen[7,8], Frank Dudbridge[9,10], Eivind Ystrom[3,4], Alexandra Havdahl[3,4,6†] & Jean-Baptiste Pingault[1,2†]

[1] Department of Clinical, Educational & Health Psychology, Division of Psychology & Language Sciences, Faculty of Brain Sciences, University College London, London, UK

[2] Social, Genetic and Developmental Psychiatry Centre, Institute of Psychiatry, Psychology & Neuroscience, King's College London, London, UK

[3] PROMENTA Research Center, Department of Psychology, University of Oslo, Oslo, Norway

[4] Nic Waals Institute, Lovisenberg Diaconal Hospital, Oslo, Norway

[5] Population Health Sciences, Bristol Medical School, University of Bristol

[6] Center for Genetic Epidemiology and Mental Health, Norwegian Institute of Public Health, Oslo, Norway

[7] NORMENT Centre, Division of Mental Health and Addiction, Oslo University Hospital & Institute of Clinical Medicine, University of Oslo, Oslo, Norway

[8] KG Jebsen Centre for Neurodevelopmental disorders, University of Oslo and Oslo University Hospital, Oslo, Norway

[9] Department of Population Health Sciences, University of Leicester, Leicester, UK

[10] NIHR Leicester Biomedical Research Centre, University of Leicester, Leicester, UK

*Correspondence: Leonard Frach, 26 Bedford Way, WC1H 0AP London, United Kingdom leonard.frach.20@ucl.ac.uk

[†]Shared senior authors

Supplementary Note

Quality Control and Imputation of Genotype Data

Poor quality variants (call rate < 0.98, minor allele frequency [MAF] < 0.005, variants that deviated from Hardy-Weinberg equilibrium (HWE) p-value < 1×10^{-6}, and Mendelian errors [ME] > 0.01 with remaining ME set to missing) were removed. Furthermore, individuals with low call rate (< 0.98), heterozygosity outliers, ME > 0.05 per family, discordant sex, ancestry outliers, and individuals of cryptically related pairs, i.e., proportion of genomes shared identical-by-descent ($\hat{\pi}$) > 0.15, were removed. Phasing, using SHAPEIT2 (1), and imputation, using IMPUTE4.1.2_r300.3 (Marchini Group), were performed using the publicly available European Genome-Phenome Archive (Study ID EGAS00001001710) Haplotype Reference Consortium release 1.1 data as a reference panel (3). Post-imputation quality control was performed on a SNP and individual level. Variants with an imputation quality score (INFO) < 0.8, MAF < 0.01, call rate < 0.95, HWE p-value < 1×10^{-6}, and ME > 0.01 (with remaining ME set to missing) were removed. Furthermore, individuals were removed if they had a call rate < 0.98, were heterozygosity outliers, ME > 0.05 per family, ancestry outliers, or cryptically related pairs ($\hat{\pi}$) > 0.15.

Description of Multiple Imputation of the Phenotypic Data

For the imputation of the age 8 years data, we included all variables used in the statistical analyses (all child and parental polygenic scores and child sex and year of birth), and we considered a range of auxiliary variables based on potential associations with missingness or the outcome, i.e., child conduct problems (see Table S3). We included auxiliary variables which were significantly associated with missingness and showed a $| r | > 0.1$ (or equivalently $R^2 > 0.01$), as recommended (4), with either the dummy variable indicating missingness or with the outcome variable (indicated by a sum score of eight items). The included auxiliary variables were maternal education during the time of pregnancy, children's behavioural problems at ages

5 and 8 years, externalising sum scores from the Child Behaviour Checklist (5) at ages 0.5, 3 and 5 years, as well as child ICD-10 diagnoses of ADHD and conduct disorder. We did not impute the age 14 years data due to the large proportion of missingness (8 035 out of 31 290 trios with genetic data also had relevant phenotype data at age 14 years, i.e., about 75% missing).

Correlation of Conduct Problems Measures Over Time

We estimated the correlation of the two conduct problems latent factors (mother-report at age 8 and self-report at age 14 years) using structural equation models in lavaan and full-information maximum likelihood. This effectively uses information from individuals with at least one conduct problem measure across both time points. The two factors were moderately correlated ($r = 0.23$, $p < 0.001$) and were similar when using complete cases only (complete phenotypic data at both time points; $r = 0.22$, $p < 0.001$).

Results Using Imputed Data

For the trio models using multiple imputed data, model fit indices were acceptable but poorer than in the dataset of complete data (CFI = 0.92, TLI = 0.89, SRMR = 0.10, RMSEA = 0.02). Results were almost identical to the results using complete data (Figure 4 in the manuscript). As in the complete data analyses, estimates for genetic transmission were significant for all polygenic scores except anxiety and were of similar effect size as in the complete data analyses (r ≥ 0.99; see Tables S5-S6, Figure 4). Hence, associations between all child polygenic scores and child conduct problems (Figure S3) were also significant after correcting for multiple testing ($| \beta | = 0.03$ to 0.12), except for the polygenic score for anxiety disorders ($\beta = 0.01$, 95 % CI [–0.02, 0.04]; see Table S6).

Furthermore, we did not find any significant genetic nurture effects using the imputed data (Table S6). In contrast to the complete data analysis, the association between the maternal polygenic score for educational attainment and child conduct problems was not significant after

adjusting for multiple testing (β = 0.03, 95% CI [0.00, 0.06]), but effect sizes were of similar magnitude and confidence intervals overlap.

Interpretation of Genetic Nurture Effects Using Maternal Polygenic Scores for Educational Attainment and Cognitive Performance

In addition, in the unadjusted model, there is no significant association between maternal polygenic scores for educational attainment and for cognitive performance and child conduct problems (β = 0.005, 95% CI [–0.02, 0.03], p = 0.691 and β = 0.017, 95% CI [–0.01, 0.04], p = 0.148, respectively). Thus, these findings may be due to suppression effects. As we observed significant negative genetic transmission using the maternal polygenic scores for educational attainment and for cognitive performance, the remaining genetic nurture effect has to be positive to reflect the observed association in the unadjusted model, which is close to zero. Another explanation for the result might be selective sampling of more highly educated parents (Table 1). Using imputed data (slightly lower levels of parental education), the observed genetic nurture effects are somewhat smaller and not significant (Figure 4). Finally, our finding might reflect a true association, as negative correlations between direct genetic effects and genetic nurture effects have been reported for conduct problems (6) and are also well-known in the animal literature using variance-component approaches (7). If this was a true association, it could also suggest that mothers with a genetic predisposition for being highly educated and for having a high cognitive performance are more likely to report higher conduct problems of their children, e.g., as they are more likely to notice problems, more likely to report them or they are more likely to interpret their children's behaviour as problematic.

Power of Polygenic Score Analyses

Our sample size consisting of 31,290 trios (of which 15,301 had available phenotype data on all items at age 8 years) was reasonably large and well-powered to detect direct genetic effects using multiple polygenic scores. However, it may still be possible that our study was

underpowered to detect small genetic nurture effects. The power for association testing using polygenic scores for psychiatric and behavioural traits to predict childhood outcomes is relatively low (e.g., in comparison to the prediction of educational outcomes using a polygenic score for educational attainment). This is also reflected in findings of lower SNP-heritability for child behavioural problems (8). The statistical power in this study was sufficient to find significant genetic transmission effects at age 8 years, and thus also sufficient to detect direct genetic effects, which are twice the size of genetic transmission (see Figure S5-S6 for power estimates for a range of sample sizes and effect sizes). Due to the substantially smaller sample size at age 14 years (N = 7,883), we could have been underpowered to detect genetic transmission and direct genetic effects of size 0.02 and 0.04, respectively. Genetic nurture effects for other behavioural traits such as education were reported to be less roughly 40% of the strength of the direct genetic effect (9,10). Hence, genetic nurture effects on conduct problems using polygenic scores might be too small to detect even in our larger sample at age 8 years. We estimated statistical power using simulations with 1,000 iterations, similar to the formula by Tubbs et al. (2020). For example, at an alpha level of 0.05, using complete data (N = 15,301 trios) and imputed data (N = 31,290 trios), we had statistical power ≥ 0.99 to detect a true direct genetic effect of 0.10 and 0.05 (e.g., close to the observed effects for the polygenic scores for ADHD and antisocial behaviour, respectively). Using an alpha level of 0.05, we had statistical power of > 0.99 to detect true genetic transmission effects of 0.05 and 0.025 (for both 15,320 trios and 31,290 trios). For a sample of 31,346 trios, the power to detect a true genetic nurture effect of strength 0.04 and 0.02 (0.4 times the direct genetic effect of 0.10 and 0.05) was > 0.99 and 0.82, respectively (Figure S5c). To detect a true genetic nurture effect of 0.04 and 0.02 in a sample of 15,320 trios, we had statistical power of 0.98 and 0.53, respectively. We further repeated the power analyses using a conservative Bonferroni-corrected alpha of 0.05/12 (i.e., ≈ 0.004), which we report in Figure S6. Statistical power for genetic transmission

and genetic nurture effects in this scenario was lower, but still sufficient so detect true genetic transmission effects of sizes 0.025 (power of > 0.99 [31,290 trios] and 0.94 [15,320 trios]) and true genetic nurture effects of size 0.04 (power of > 0.99 [31,290 trios] and 0.89 [15,320 trios]).

Supplementary Figures

Figure S1

Convergence Plots of the Multiple Imputation of Phenotype Data

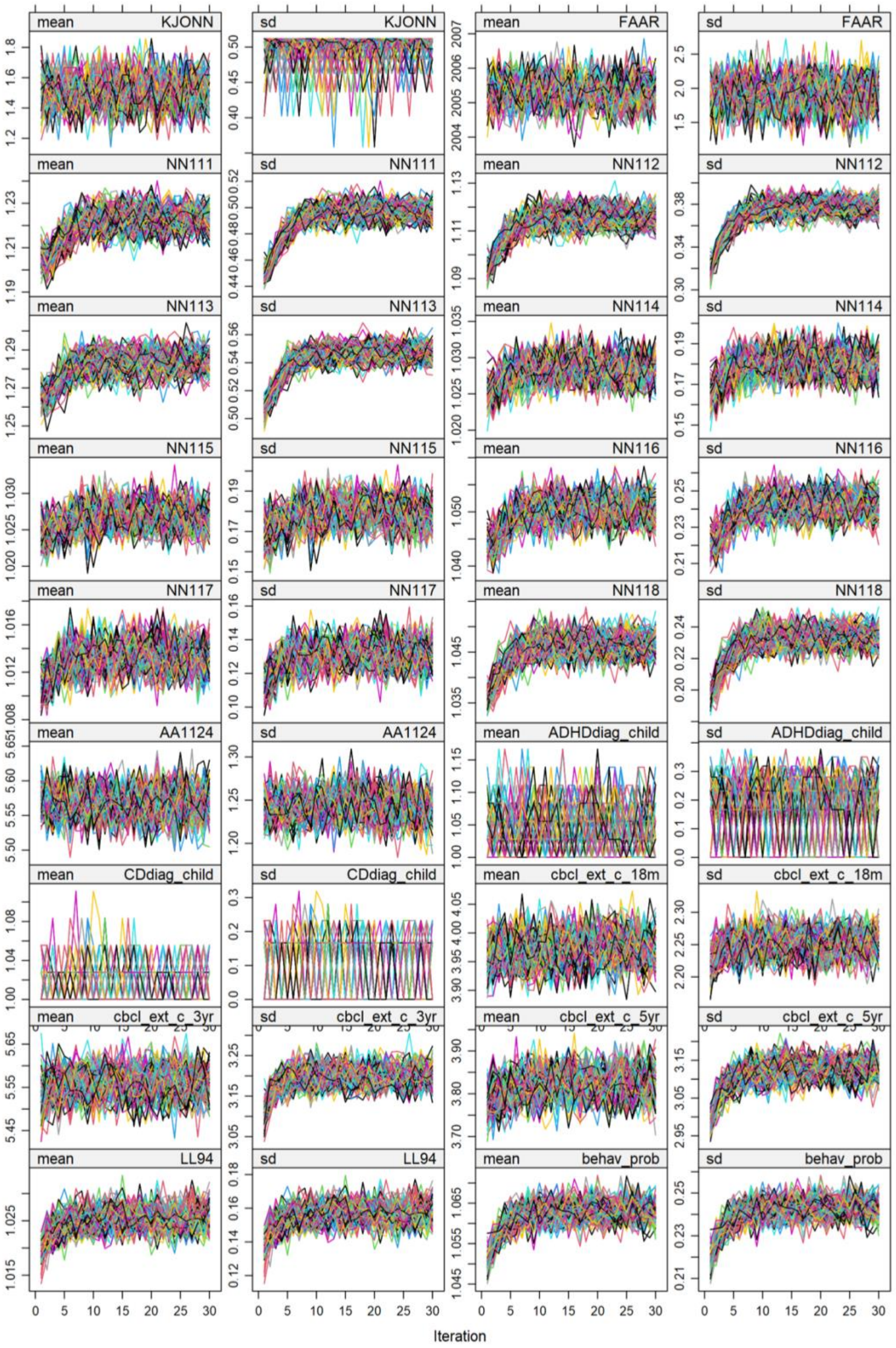

Note. Variables NN111 to NN118 indicate the 8 items of the conduct disorder subscale. AA1124 = maternal education (during pregnancy), FAAR = year of birth, KJONN = child sex, LL94 = behavioural problems (5 years), behav_prob = behavioural problems (8 years)

Figure S2

Distributions of the Observed and Imputed Phenotype Data for Continuous Variables Used in the Imputation

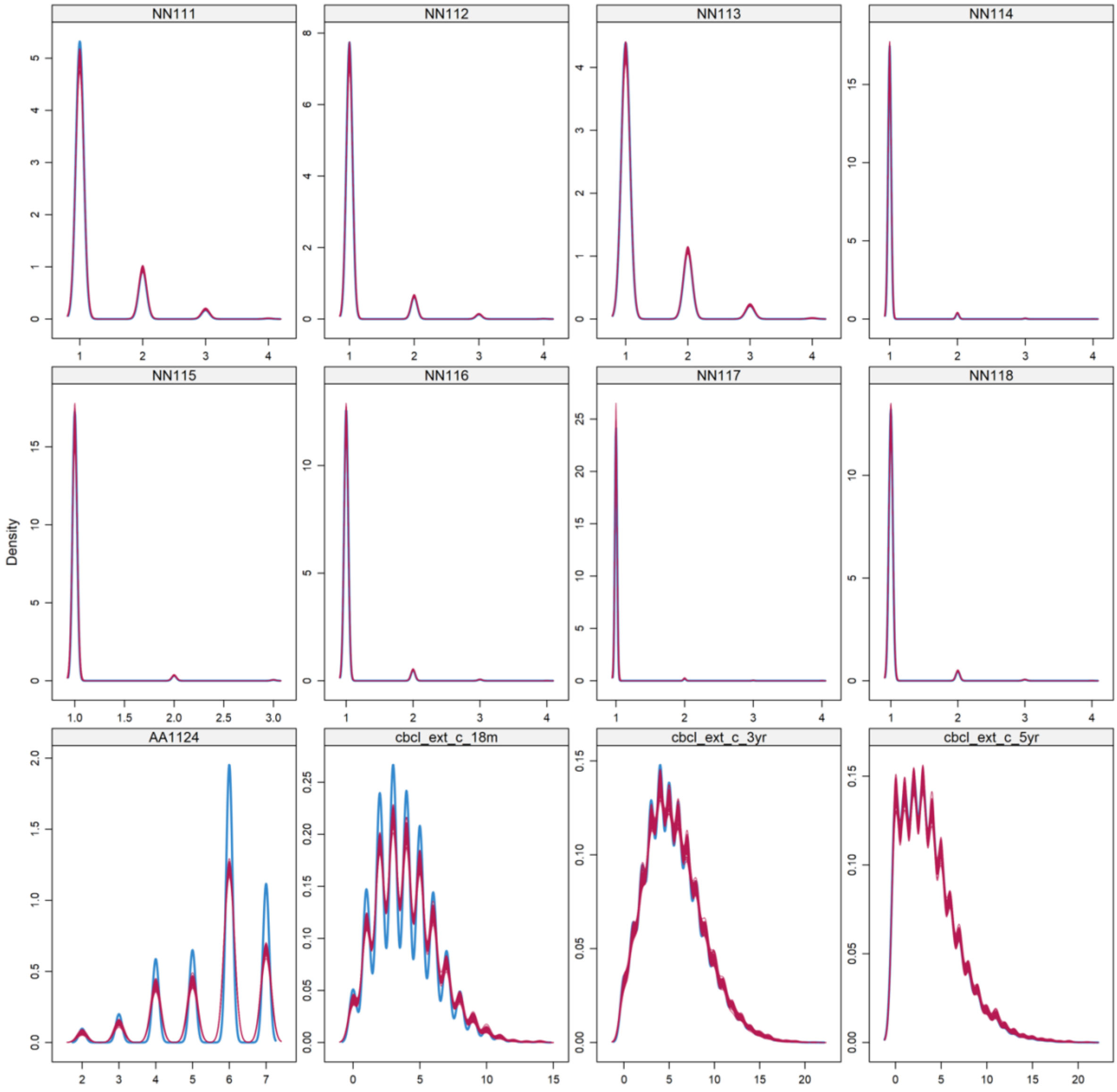

Figure S3

Direct Genetic Effects at Age 8 Years

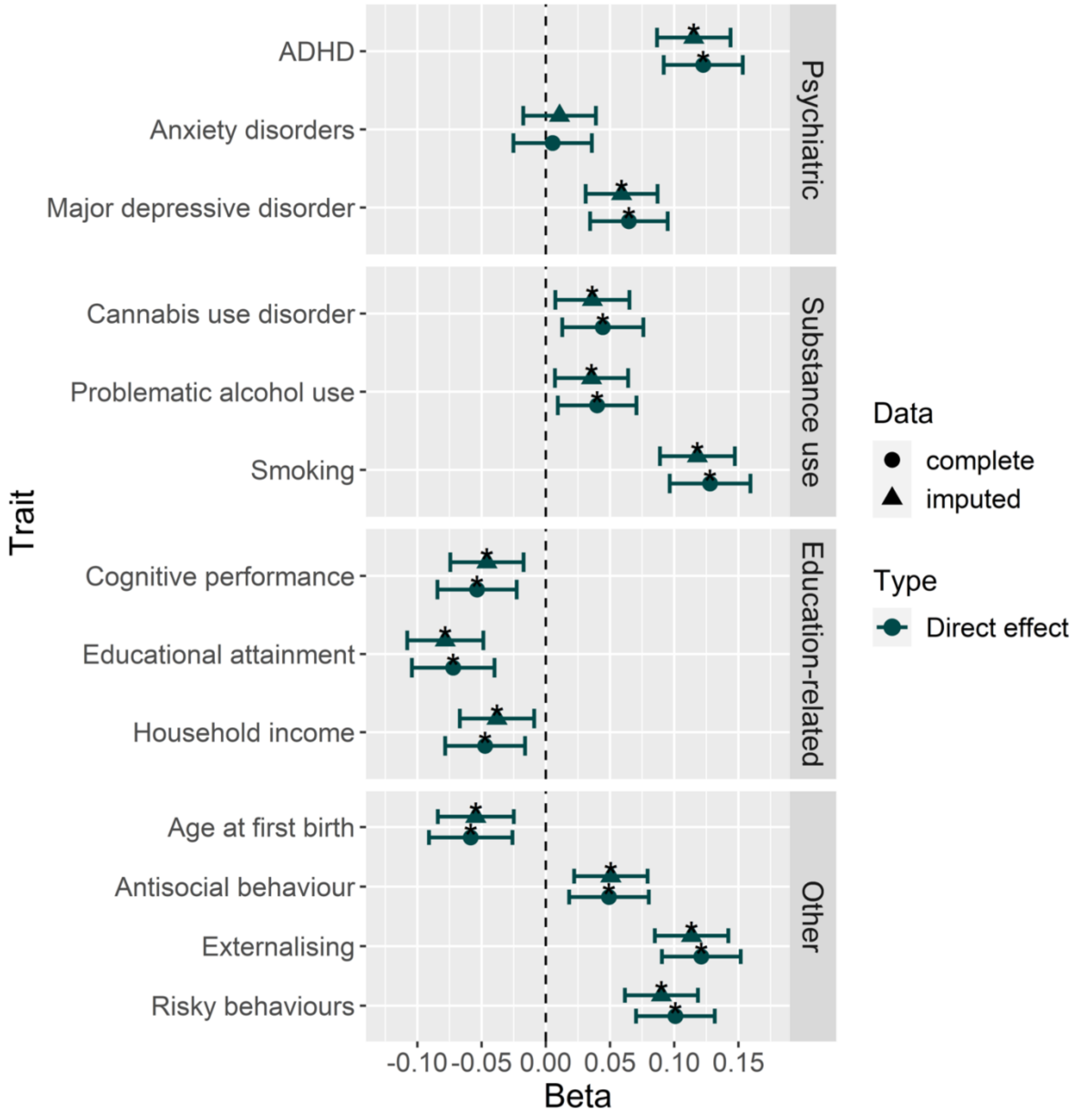

Note. Direct genetic effects are estimated from the trio models and reflect the pathway β_C in Figure 2 of the manuscript.

Figure S4

Direct Genetic Effects at Age 14 Years

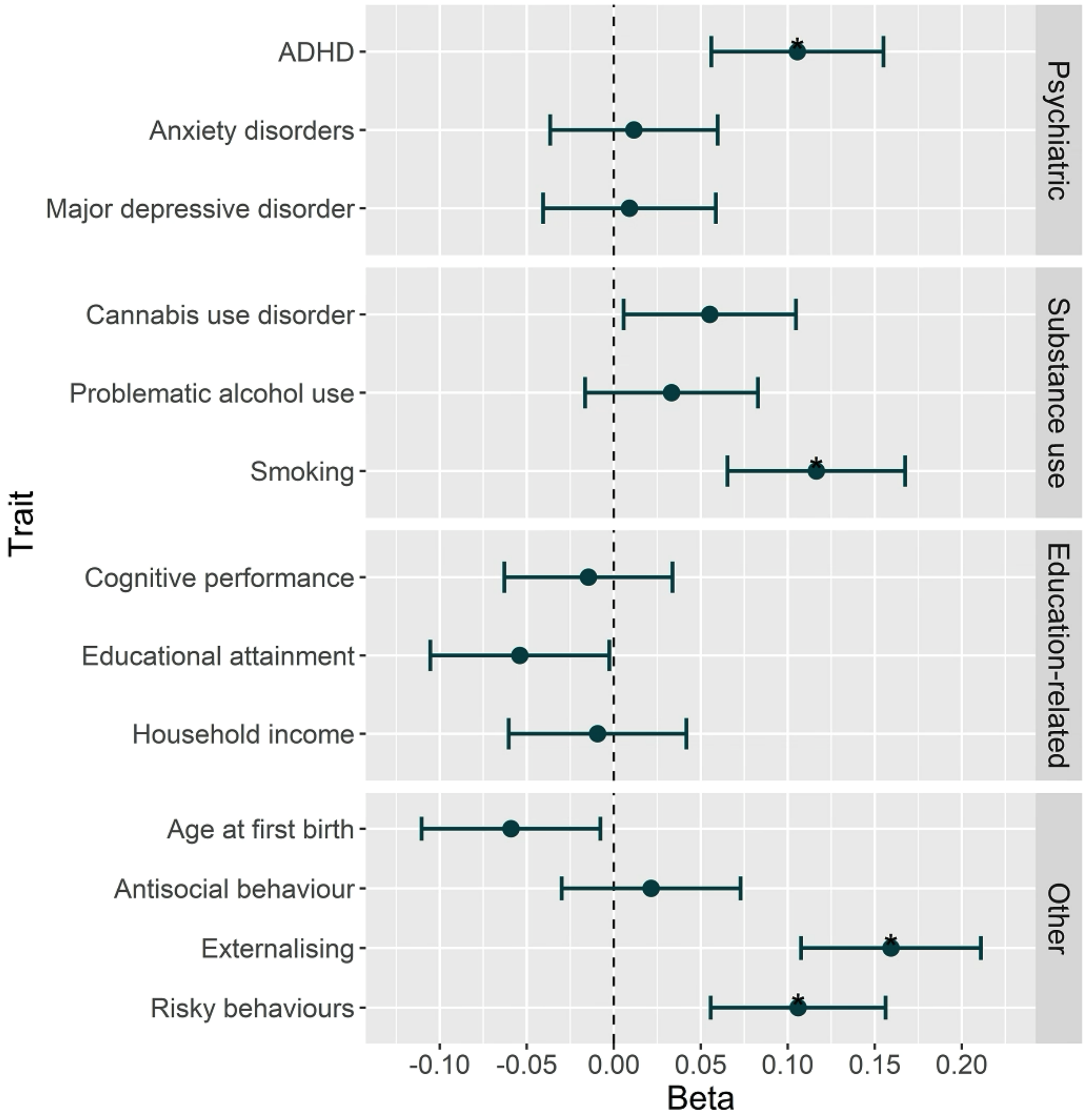

Note. Direct genetic effects are estimated from the trio models and reflect the pathway β_C in Figure 2 of the manuscript.

Figure S5

Direct Genetic Effect Estimates in Trio Models vs Univariate Models Using Complete Data

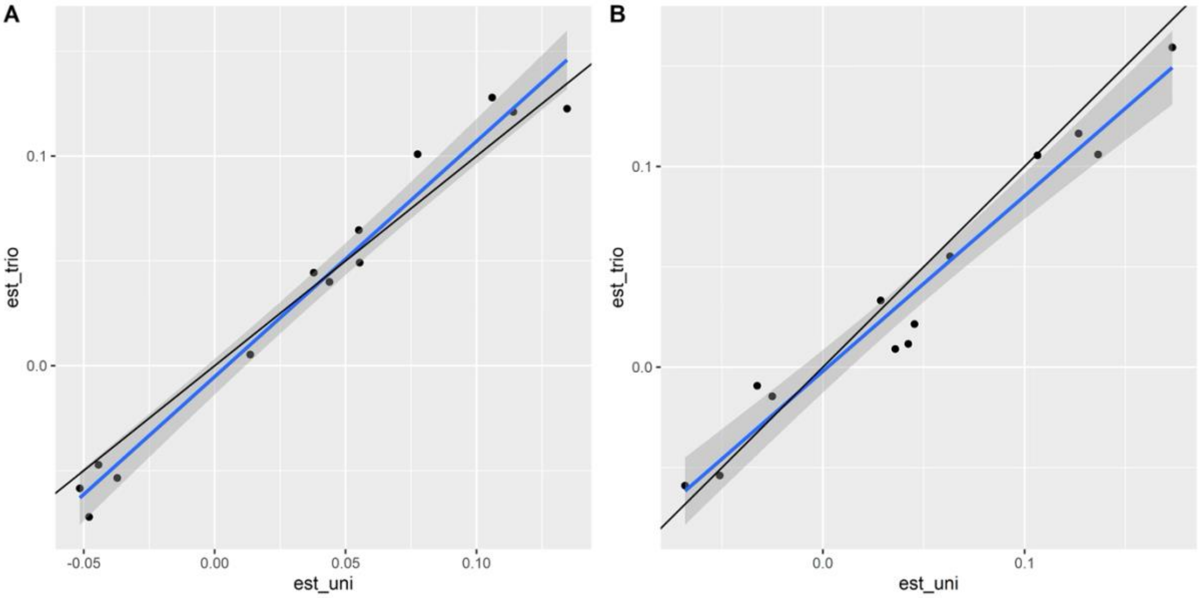

Note. Correlations between beta estimates of the trio models with the unadjusted univariate models (which were done for comparison). The black diagonal shows a line with a slope of 1. **A** Correlation for beta estimates of child polygenic score sat age 8 years. **B** Correlation for beta estimates of child polygenic scores at age 14 years.

Figure S6

Direct Genetic Effect Estimates in Trio Models vs Univariate Models Using Imputed Data

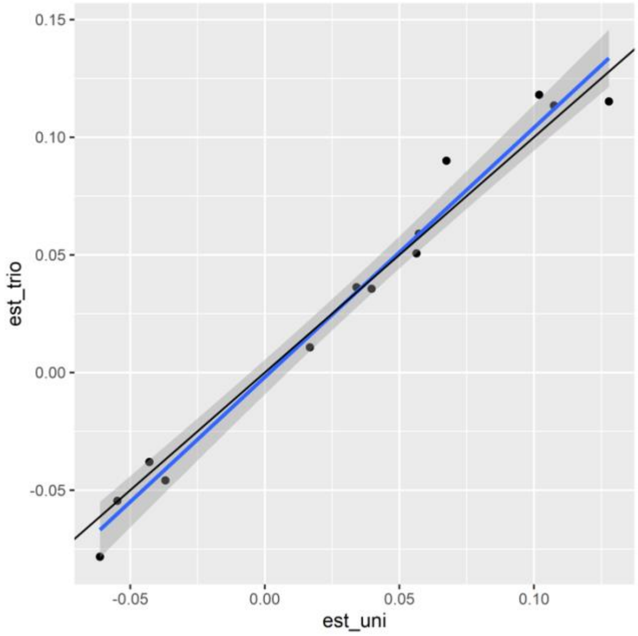

Note. Correlations between beta estimates of the trio models with the unadjusted univariate models for imputed data at age 8 years (which were done for comparison). The black diagonal shows a line with a slope of 1.

Figure S7

Results From Power Analyses for Trio Models Using Polygenic Scores

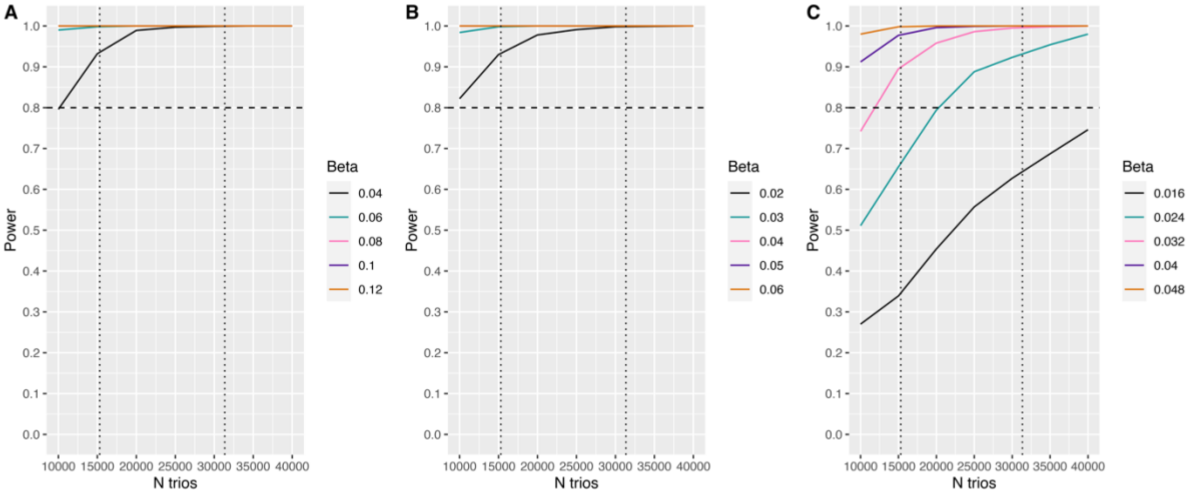

Note. Power analyses for trio models using polygenic scores were performed using 1,000 iterations with an α level of 0.05. **A** power estimates for child (direct genetic) effects. **B** power estimates for genetic transmission effects (0.5 times the size of the direct genetic effects). **C** power estimates for genetic nurture effects, where the genetic nurture effect is 0.4 times the size of the direct genetic effect. The dotted vertical lines indicate the sample size in our analyses.

Figure S8

Results From Power Analyses (Bonferroni-corrected)

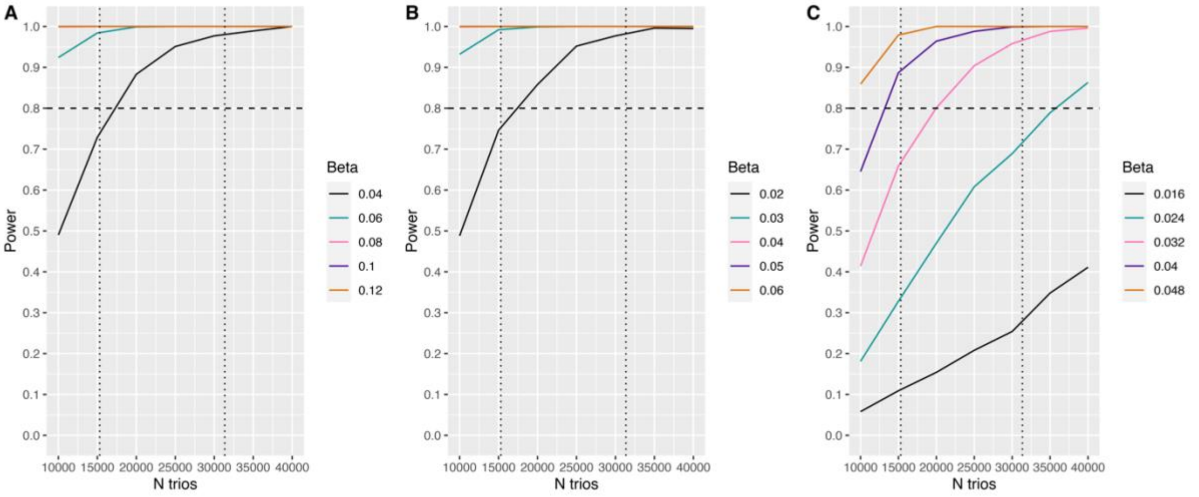

Note. Power analyses for trio models using polygenic scores were performed using 1,000 iterations with an α level of 0.05/12 (i.e., ≈ 0.004). **A** power estimates for child (direct genetic) effects. **B** power estimates for genetic transmission effects (0.5 times the size of the direct genetic effects). **C** power estimates for genetic nurture effects, where the genetic nurture effect is 0.4 times the size of the direct genetic effect. The dotted vertical lines indicate the sample size in our analyses.

Supplementary Tables

Table S1

STROBE Statement—Checklist of items that should be included in reports of cohort studies

	Item No	Recommendation
**Title and abstract**	1	(*a*) Indicate the study's design with a commonly used term in the title or the abstract ***(pages 1–2)***
		(*b*) Provide in the abstract an informative and balanced summary of what was done and what was found ***(page 2)***
**Introduction**		
Background/rationale	2	Explain the scientific background and rationale for the investigation being reported ***(pages 3–6)***
Objectives	3	State specific objectives, including any prespecified hypotheses ***(pages 5–6)***
**Methods**		
Study design	4	Present key elements of study design early in the paper ***(pages 6–7)***
Setting	5	Describe the setting, locations, and relevant dates, including periods of recruitment, exposure, follow-up, and data collection ***(pages 6–7)***
Participants	6	(*a*) Give the eligibility criteria, and the sources and methods of selection of participants. Describe methods of follow-up ***(pages 6–7)***
		(*b*) For matched studies, give matching criteria and number of exposed and unexposed
Variables	7	Clearly define all outcomes, exposures, predictors, potential confounders, and effect modifiers. Give diagnostic criteria, if applicable ***(pages 7–9)***
Data sources/ measurement	8*	For each variable of interest, give sources of data and details of methods of assessment (measurement). Describe comparability of assessment methods if there is more than one group ***(pages 7–9)***
Bias	9	Describe any efforts to address potential sources of bias ***(pages 8–9, 12, 19, 20–21, Supplementary materials)***
Study size	10	Explain how the study size was arrived at ***(pages 6–8, 12)***
Quantitative variables	11	Explain how quantitative variables were handled in the analyses. If applicable, describe which groupings were chosen and why ***(pages 9–11)***
Statistical methods	12	(*a*) Describe all statistical methods, including those used to control for confounding ***(pages 9–12)***
		(*b*) Describe any methods used to examine subgroups and interactions
		(*c*) Explain how missing data were addressed ***(page 12, Supplementary materials)***
		(*d*) If applicable, explain how loss to follow-up was addressed
		(*e*) Describe any sensitivity analyses ***(pages 10, 12, Supplementary materials)***
**Results**		
Participants	13*	(a) Report numbers of individuals at each stage of study—eg numbers potentially eligible, examined for eligibility, confirmed eligible, included in the study, completing follow-up, and analysed ***(page 12)***
		(b) Give reasons for non-participation at each stage ***(pages 6–8, 12, Table 1)***
		(c) Consider use of a flow diagram
Descriptive data	14*	(a) Give characteristics of study participants (eg demographic, clinical, social) and information on exposures and potential confounders ***(Table 1)***
		(b) Indicate number of participants with missing data for each variable of interest ***(Table 1, page 12)***
		(c) Summarise follow-up time (eg, average and total amount)
Outcome data	15*	Report numbers of outcome events or summary measures over time ***(page 7)***

Table S1 continued

Main results	16	(*a*) Give unadjusted estimates and, if applicable, confounder-adjusted estimates and their precision (eg, 95% confidence interval). Make clear which confounders were adjusted for and why they were included ***(pages 9–10, 15–16, Tables S4–S8)***
		(*b*) Report category boundaries when continuous variables were categorized
		(*c*) If relevant, consider translating estimates of relative risk into absolute risk for a meaningful time period
Other analyses	17	Report other analyses done—eg analyses of subgroups and interactions, and sensitivity analyses ***(page 12, Supplementary materials)***
**Discussion**		
Key results	18	Summarise key results with reference to study objectives ***(pages 16–17)***
Limitations	19	Discuss limitations of the study, taking into account sources of potential bias or imprecision. Discuss both direction and magnitude of any potential bias ***(pages 19–22)***
Interpretation	20	Give a cautious overall interpretation of results considering objectives, limitations, multiplicity of analyses, results from similar studies, and other relevant evidence ***(pages 17–20)***
Generalisability	21	Discuss the generalisability (external validity) of the study results ***(pages 20–21)***
**Other information**		
Funding	22	Give the source of funding and the role of the funders for the present study and, if applicable, for the original study on which the present article is based ***(page 22)***

*Give information separately for exposed and unexposed groups.

Table S2

Reported SNP Heritability Estimates, Standard Errors, and Z-Scores for Each GWAS Outcome

Domain	GWAS	Publication	h^2_{SNP}	*SE*	*Z*
Psychopathology	ADHD	(12)	0.217	0.014	15.48
	Anxiety disorder	(13)	0.165	0.011	15.23
	Depression	(14)	0.100	0.004	25.62
Education-related factors	Cognitive performance	(15)	0.192	0.006	31.51
	Educational attainment[1]	(15)	0.147	0.009	16.33
	Income	(16)	0.062	0.003	20.03
Substance use (disorders)	Cannabis use disorder	(17)	0.121	0.011	11.00
	AUDIT-P	(18)	0.059	0.005	12.29
	Smoking	(Wootton et al., 2020)	0.094	0.003	29.47
Other risky behaviours	Age at first birth	(19)	0.060	0.003	20.00
	Antisocial behaviour	(20)	0.084	0.012	7.00
	Risky behaviours	(21)	0.156	0.004	39.00

Note. [1]excluding MoBa and 23andMe participants; Reported SNP heritability estimates in the original GWAS represent the SNP heritability estimated using LD score regression (22). Summary statistics from the externalising GWAS excluding 23andMe data (23,24), which here were used as an additional analysis, did not report SNP heritability estimates as they performed Genomic SEM on a latent factor of externalising. However, prediction of the externalising polygenic score (which can be conceptualised as the lower bound of the SNP heritability) in the original validation samples was >8%, suggesting that the SNP heritability was well above our threshold of 0.05.

AUDIT-P = Alcohol use disorder identification test (problematic use subscale).

Table S3

Auxiliary Variables Considered for Multiple Imputation

Variable	Measurement time point	Used for imputation
Maternal age	Time of birth	no
Paternal age	Time of birth	no
Health region (mother's residence)	Time of birth	no
Mother's municipality of residence	Time of birth	no
Mother's county of residence	Time of birth	no
Mother's marital status	Time of birth	no
Number of previous deliveries	Time of birth	no
Maternal smoking	Start of pregnancy	no
Cigarettes per day (mother)	Start of pregnancy	no
Length of gestation (weeks)	Time of birth	no
Child weight in grams	Time of birth	no
Maternal diabetes (pre-/gestational)	Prior to pregnancy	no
Hypertensive condition	Time of birth	no
Preeclampsia	Time of birth	no
Birth complications	Time of birth	no
ICD-10 F90 Hyperkinetic disorders	Ever diagnosed	**yes**
F91 conduct disorders	Ever diagnosed	**yes** (F91+F92)
F92 Mixed disorders of conduct & emotions	Ever diagnosed	**yes** ((F91+F92)
F90 Hyperkinetic disorders (mother)	Ever diagnosed	no
F91 conduct disorders (mother)	Ever diagnosed	no
F92 (mother)	Ever diagnosed	no
F10-F19 Mental and behavioural disorders due to psychoactive substance use (mother)	Ever diagnosed	no
F32-F34 Depression (mother)	Ever diagnosed	no
F60.2 Dissocial personality disorder (mother)	Ever diagnosed	no
F90 Hyperkinetic disorders (father)	Ever diagnosed	no
F91 conduct disorders (father)	Ever diagnosed	no
F92 (father)	Ever diagnosed	no

Table S3 continued

Variable	Measurement time point	Used for imputation
F10-F19 Mental and behavioural disorders due to psychoactive substance use (father)	Ever diagnosed	no
F32-F34 Depression (father)	Ever diagnosed	no
F60.2 Dissocial personality disorder (father)	Ever diagnosed	no
Maternal education	15th week of gestation	**yes**
Maternal income	15th week of gestation	no
Maternal BMI	15th week of gestation	no
Paternal education	15th week of gestation	no
Paternal income	15th week of gestation	no
Intake of testosterone products	30th week of gestation	no
Child's mood and temperament	6 months	no
Child behaviour checklist (externalising)	18 months	**yes**
Coping with financial situations (mother)	18 months	no
Living with child's father	18 months	no
Child behaviour checklist (externalising)	3 years	**yes**
Living with child's father	3 years	no
Child behaviour checklist (externalising)	5 years	**yes**
Behavioural problems (child)	5 years	**yes**
Behavioural problems (child)	8 years	**yes**
Maternal education	8 years	no

Table S4

Bivariate Correlations Between Each Polygenic Score on the Child Level

**PGS**	ADHD	Anxiety	MDD	CUD	AUDIT-P	Smoking	CogPerf	EA	Income	AFB	ASB	Risk	EXT
ADHD	1	0.11	0.25	0.19	0.04	0.30	–0.20	–0.29	–0.18	–0.31	0.24	0.16	0.34
Anxiety	0.11	1	0.41	0.07	0.10	0.12	–0.05	–0.06	–0.10	–0.10	0.07	0.05	0.13
MDD	0.25	0.41	1	0.11	0.09	0.22	–0.08	–0.13	–0.15	–0.20	0.12	0.11	0.22
CUD	0.19	0.07	0.11	1	0.07	0.21	–0.09	–0.15	–0.08	–0.18	0.13	0.17	0.26
AUDIT-P	0.04	0.10	0.09	0.07	1	0.15	0.04	0.01	0.02	–0.01	0.03	0.29	0.24
Smoking	0.30	0.12	0.22	0.21	0.15	1	–0.17	–0.34	–0.22	–0.33	0.17	0.43	0.59
CognPerf	–0.20	–0.05	–0.08	–0.09	0.04	–0.17	1	0.50	0.36	0.28	–0.07	0.04	–0.14
EA	–0.29	–0.06	–0.13	–0.15	0.01	–0.34	0.50	1	0.48	0.49	–0.13	<0.01	–0.24
Income	–0.18	–0.10	–0.15	–0.08	0.02	–0.22	0.36	0.48	1	0.31	–0.09	0.09	–0.09
AFB	–0.31	–0.10	–0.20	–0.18	–0.01	–0.33	0.28	0.49	0.31	1	–0.14	–0.07	–0.32
ASB	0.24	0.07	0.12	0.13	0.03	0.17	–0.07	–0.13	–0.09	–0.14	1	0.11	0.20
Risk	0.16	0.05	0.11	0.17	0.29	0.43	0.04	<0.01	0.09	–0.07	0.11	1	0.70
EXT	0.34	0.13	0.22	0.26	0.24	0.59	–0.14	–0.24	–0.09	–0.32	0.20	0.70	1

Note. ASB = antisocial behaviour, AFB = age at first birth, EXT = externalising behaviours, MDD = Major depressive disorder, CognPerf = Cognitive performance, EA = Educational attainment, CUD = Cannabis use disorder, Risk = Risky behaviours

Table S5

Main Results From the Trio Models Using Complete Data at Age 8 Years

Effect	PGS	*β*	*SE*	*Z*	*p*-value	CI lower	CI upper	*q*-value
**Child (direct)**	ADHD	0.123	0.016	7.876	3.3×10^{-15}	0.092	0.153	**2.2×10^{-14}**
	Anxiety	0.005	0.016	0.341	0.733	–0.025	0.036	0.733
	MDD	0.065	0.015	4.205	2.6×10^{-5}	0.034	0.095	**5.7×10^{-5}**
	CUD	0.044	0.016	2.757	0.006	0.013	0.076	**0.007**
	AUDIT-P	0.040	0.016	2.562	0.010	0.009	0.071	**0.011**
	Smoking	0.128	0.016	8.026	1.1×10^{-15}	0.096	0.159	**1.4×10^{-14}**
	CognPerf	–0.054	0.016	–3.405	0.001	–0.084	–0.023	**0.001**
	EA	–0.072	0.016	–4.402	1.1×10^{-5}	–0.104	–0.040	**2.8×10^{-5}**
	Income	–0.047	0.016	–2.980	0.003	–0.078	–0.016	**0.004**
	AgeFirstBirth	–0.058	0.017	–3.534	4.1×10^{-4}	–0.091	–0.026	**0.001**
	ASB	0.049	0.016	3.114	0.002	0.018	0.080	**0.003**
	Risk	0.101	0.016	6.475	9.5×10^{-11}	0.070	0.132	**3.1×10^{-10}**
	Externalising	0.121	0.016	7.788	6.9×10^{-15}	0.090	0.152	**3.0×10^{-14}**
**Genetic transmission**	ADHD_f	0.059	0.008	7.840	4.4×10^{-15}	0.044	0.074	**2.9×10^{-14}**
	ADHD_m	0.059	0.008	7.841	4.4×10^{-15}	0.044	0.074	**2.9×10^{-14}**
	Anxiety_f	0.003	0.008	0.341	0.733	–0.013	0.018	0.733
	Anxiety_m	0.003	0.008	0.341	0.733	–0.012	0.018	0.733
	MDD_f	0.031	0.008	4.200	2.7×10^{-5}	0.017	0.046	**5.8×10^{-5}**
	MDD_m	0.032	0.008	4.200	2.7×10^{-5}	0.017	0.046	**5.8×10^{-5}**
	CUD_f	0.022	0.008	2.756	0.006	0.006	0.038	**0.007**
	CUD_m	0.022	0.008	2.756	0.006	0.006	0.037	**0.007**
	AUDIT-P_f	0.020	0.008	2.561	0.010	0.005	0.035	**0.011**
	AUDIT-P_m	0.020	0.008	2.561	0.010	0.005	0.035	**0.011**
	Smoking_f	0.063	0.008	7.990	1.3×10^{-15}	0.048	0.079	**1.7×10^{-14}**
	Smoking_m	0.063	0.008	7.992	1.3×10^{-15}	0.048	0.079	**1.7×10^{-14}**
	CognPerf_f	–0.026	0.008	–3.403	0.001	–0.041	–0.011	**0.001**
	CognPerf_m	–0.026	0.008	–3.403	0.001	–0.041	–0.011	**0.001**
	EA_f	–0.036	0.008	–4.397	1.1×10^{-5}	–0.052	–0.020	**2.9×10^{-5}**
	EA_m	–0.035	0.008	–4.396	1.1×10^{-5}	–0.050	–0.019	**2.9×10^{-5}**
	Income_f	–0.023	0.008	–2.978	0.003	–0.039	–0.008	**0.004**
	Income_m	–0.023	0.008	–2.978	0.003	–0.038	–0.008	**0.004**
	AgeFirstBirth_f	–0.029	0.008	–3.531	4.1×10^{-4}	–0.045	–0.013	**0.001**
	AgeFirstBirth_m	–0.028	0.008	–3.531	4.1×10^{-4}	–0.044	–0.013	**0.001**
	ASB_f	0.024	0.008	3.112	0.002	0.009	0.039	**0.003**
	ASB_m	0.024	0.008	3.112	0.002	0.009	0.040	**0.003**
	Risk_f	0.050	0.008	6.456	1.1×10^{-10}	0.035	0.065	**3.5×10^{-10}**
	Risk_m	0.051	0.008	6.457	1.1×10^{-10}	0.035	0.066	**3.5×10^{-10}**
	Externalising_f	0.059	0.008	7.756	8.7×10^{-15}	0.044	0.075	**3.8×10^{-14}**
	Externalising_m	0.060	0.008	7.755	8.9×10^{-15}	0.045	0.076	**3.8×10^{-14}**

Table S5 continued

Effect	PGS	*β*	*SE*	*Z*	*p*-value	CI lower	CI upper	*q*-value
**Genetic nurture**	ADHD_f	0.008	0.014	0.590	0.555	–0.019	0.035	0.912
	ADHD_m	0.015	0.014	1.114	0.265	–0.012	0.042	0.766
	Anxiety_f	0.009	0.014	0.662	0.508	–0.018	0.036	0.912
	Anxiety_m	0.008	0.014	0.549	0.583	–0.020	0.035	0.912
	MDD_f	–0.020	0.014	–1.512	0.131	–0.047	0.006	0.679
	MDD_m	0.001	0.014	0.067	0.947	–0.026	0.028	0.966
	CUD_f	–0.003	0.014	–0.245	0.807	–0.030	0.024	0.912
	CUD_m	–0.010	0.014	–0.723	0.469	–0.037	0.017	0.912
	AUDIT-P_f	0.005	0.014	0.374	0.709	–0.022	0.032	0.912
	AUDIT-P_m	0.003	0.014	0.246	0.806	–0.023	0.030	0.912
	Smoking_f	–0.025	0.014	–1.834	0.067	–0.052	0.002	0.434
	Smoking_m	–0.016	0.014	–1.185	0.236	–0.043	0.011	0.766
	CognPerf_f	–0.012	0.014	–0.850	0.395	–0.039	0.015	0.912
	CognPerf_m	0.044	0.014	3.183	0.001	0.017	0.071	**0.023**
	EA_f	0.001	0.014	0.042	0.966	–0.026	0.028	0.966
	EA_m	0.044	0.014	3.129	0.002	0.017	0.072	**0.023**
	Income_f	0.002	0.014	0.138	0.890	–0.025	0.029	0.964
	Income_m	0.004	0.014	0.311	0.756	–0.022	0.031	0.912
	AgeFirstBirth_f	–0.004	0.014	–0.294	0.769	–0.031	0.023	0.912
	AgeFirstBirth_m	0.017	0.014	1.237	0.216	–0.010	0.045	0.766
	ASB_f	0.004	0.014	0.317	0.752	–0.023	0.031	0.912
	ASB_m	0.007	0.014	0.531	0.596	–0.020	0.035	0.912
	Risk_f	–0.019	0.014	–1.402	0.161	–0.046	0.008	0.698
	Risk_m	–0.028	0.014	–2.012	0.044	–0.055	–0.001	0.383
	Externalising_f	–0.005	0.014	–0.347	0.729	–0.031	0.022	0.912
	Externalising_m	–0.009	0.014	–0.628	0.530	–0.035	0.018	0.912

Note. Genetic transmission effects are equal for mothers and fathers. Genetic nurture effects: f = father, m = mother. *q*-values in bold indicate associations with $q < .05$ (FDR). CI lower = lower bound of the 95% confidence interval, CI upper = upper bound of the 95% confidence interval.

Table S6

Results From the Trio Models Using Imputed Data at Age 8 Years

Effect	PGS	*β*	*SE*	*t*	*p*-value	CI lower	CI upper	*q*-value
**Child (direct)**	ADHD	0.115	0.015	7.960	$1.0{\times}10^{-14}$	0.087	0.144	**$7.8{\times}10^{-14}$**
	Anxiety	0.011	0.014	0.743	0.457	–0.018	0.039	0.457
	MDD	0.059	0.014	4.141	$4.0{\times}10^{-5}$	0.031	0.087	**$8.6{\times}10^{-5}$**
	CUD	0.036	0.015	2.466	0.014	0.007	0.065	**0.016**
	AUDIT-P	0.036	0.015	2.452	0.015	0.007	0.064	**0.016**
	Smoking	0.118	0.015	7.978	$1.2{\times}10^{-14}$	0.089	0.147	**$7.8{\times}10^{-14}$**
	CognPerf	–0.046	0.015	–3.153	0.002	–0.074	–0.017	**0.002**
	EA	–0.078	0.015	–5.190	$3.0{\times}10^{-7}$	–0.108	–0.049	**$7.8{\times}10^{-7}$**
	Income	–0.038	0.015	–2.575	0.010	–0.067	–0.009	**0.013**
	AgeFirstBirth	–0.055	0.015	–3.618	$3.3{\times}10^{-4}$	–0.084	–0.025	**0.001**
	ASB	0.051	0.015	3.478	0.001	0.022	0.079	**0.001**
	Risk	0.090	0.015	6.221	$9.8{\times}10^{-10}$	0.062	0.118	**$3.2{\times}10^{-9}$**
	Externalising	0.114	0.015	7.820	$2.7{\times}10^{-14}$	0.085	0.142	**$1.2{\times}10^{-13}$**
**Genetic transmission**	ADHD_f	0.056	0.007	7.923	$1.3{\times}10^{-14}$	0.042	0.070	**$9.7{\times}10^{-14}$**
	ADHD_m	0.057	0.007	7.925	$1.3{\times}10^{-14}$	0.042	0.071	**$9.6{\times}10^{-14}$**
	Anxiety_f	0.005	0.007	0.743	0.458	–0.009	0.019	0.458
	Anxiety_m	0.005	0.007	0.743	0.458	–0.009	0.019	0.458
	MDD_f	0.029	0.007	4.132	$4.1{\times}10^{-5}$	0.015	0.042	**$8.9{\times}10^{-5}$**
	MDD_m	0.029	0.007	4.133	$4.1{\times}10^{-5}$	0.015	0.043	**$8.9{\times}10^{-5}$**
	CUD_f	0.018	0.007	2.465	0.014	0.004	0.032	**0.016**
	CUD_m	0.018	0.007	2.465	0.014	0.004	0.032	**0.016**
	AUDIT-P_f	0.018	0.007	2.451	0.015	0.004	0.032	**0.016**
	AUDIT-P_m	0.018	0.007	2.451	0.015	0.004	0.032	**0.016**
	Smoking_f	0.058	0.007	7.945	$1.5{\times}10^{-14}$	0.044	0.072	**$9.7{\times}10^{-14}$**
	Smoking_m	0.059	0.007	7.946	$1.5{\times}10^{-14}$	0.044	0.073	**$9.6{\times}10^{-14}$**
	CognPerf_f	–0.023	0.007	–3.152	0.002	–0.037	–0.009	**0.002**
	CognPerf_m	–0.022	0.007	–3.152	0.002	–0.036	–0.008	**0.002**
	EA_f	–0.039	0.007	–5.180	$3.2{\times}10^{-7}$	–0.053	–0.024	**$8.2{\times}10^{-7}$**
	EA_m	–0.038	0.007	–5.180	$3.2{\times}10^{-7}$	–0.053	–0.024	**$8.3{\times}10^{-7}$**
	Income_f	–0.019	0.007	–2.574	0.010	–0.033	–0.004	**0.013**
	Income_m	–0.019	0.007	–2.574	0.010	–0.033	–0.004	**0.013**
	AgeFirstBirth_f	–0.027	0.007	–3.615	$3.3{\times}10^{-4}$	–0.042	–0.012	**0.001**
	AgeFirstBirth_m	–0.027	0.007	–3.615	$3.3{\times}10^{-4}$	–0.042	–0.012	**0.001**
	ASB_f	0.025	0.007	3.474	0.001	0.011	0.039	**0.001**
	ASB_m	0.025	0.007	3.474	0.001	0.011	0.039	**0.001**
	Risk_f	0.044	0.007	6.207	$1.1{\times}10^{-9}$	0.030	0.058	**$3.4{\times}10^{-9}$**
	Risk_m	0.045	0.007	6.208	$1.1{\times}10^{-9}$	0.031	0.059	**$3.4{\times}10^{-9}$**
	Externalising_f	0.056	0.007	7.791	$3.3{\times}10^{-14}$	0.042	0.070	**$1.4{\times}10^{-13}$**
	Externalising_m	0.056	0.007	7.792	$3.3{\times}10^{-14}$	0.042	0.071	**$1.4{\times}10^{-13}$**

Table S6 continued

Effect	PGS	*β*	*SE*	*t*	*p*-value	CI lower	CI upper	*q*-value
**Genetic nurture**	ADHD_f	0.007	0.013	0.560	0.576	–0.018	0.032	0.898
	ADHD_m	0.018	0.013	1.430	0.153	–0.007	0.043	0.664
	Anxiety_f	0.001	0.013	0.076	0.939	–0.024	0.026	0.977
	Anxiety_m	0.011	0.013	0.893	0.372	–0.014	0.036	0.898
	MDD_f	–0.013	0.012	–1.027	0.305	–0.037	0.012	0.898
	MDD_m	0.009	0.013	0.735	0.462	–0.016	0.034	0.898
	CUD_f	0.001	0.013	0.092	0.927	–0.024	0.026	0.977
	CUD_m	–0.006	0.013	–0.448	0.654	–0.030	0.019	0.898
	AUDIT-P_f	0.003	0.013	0.261	0.794	–0.022	0.028	0.898
	AUDIT-P_m	0.005	0.013	0.406	0.685	–0.020	0.030	0.898
	Smoking_f	–0.023	0.013	–1.791	0.074	–0.048	0.002	0.386
	Smoking_m	–0.008	0.013	–0.641	0.522	–0.033	0.017	0.898
	CognPerf_f	–0.013	0.013	–1.000	0.317	–0.038	0.012	0.898
	CognPerf_m	0.030	0.013	2.405	0.016	0.006	0.055	0.230
	EA_f	0.000	0.013	0.028	0.978	–0.025	0.025	0.978
	EA_m	0.031	0.013	2.380	0.018	0.005	0.056	0.230
	Income_f	–0.005	0.013	–0.357	0.721	–0.030	0.020	0.898
	Income_m	–0.005	0.013	–0.384	0.701	–0.030	0.020	0.898
	AgeFirstBirth_f	–0.007	0.013	–0.567	0.571	–0.032	0.018	0.898
	AgeFirstBirth_m	0.007	0.013	0.523	0.601	–0.018	0.032	0.898
	ASB_f	0.004	0.013	0.326	0.745	–0.021	0.029	0.898
	ASB_m	0.007	0.013	0.569	0.570	–0.018	0.032	0.898
	Risk_f	–0.023	0.013	–1.789	0.074	–0.047	0.002	0.386
	Risk_m	–0.023	0.013	–1.804	0.072	–0.048	0.002	0.386
	Externalising_f	–0.008	0.013	–0.627	0.531	–0.033	0.017	0.898
	Externalising_m	–0.004	0.013	–0.291	0.771	–0.029	0.021	0.898

Note. Genetic transmission effects are equal for mothers and fathers. Genetic nurture effects: f = father, m = mother. *q*-values in bold indicate associations with $q < .05$ (FDR). CI lower = lower bound of the 95% confidence interval, CI upper = upper bound of the 95% confidence interval.

Table S7

Results From the Trio Models Using Complete Data at Age 14 Years

Effect	PGS	*β*	*SE*	*Z*	*p*-value	CI lower	CI upper	*q*-value
**Child (direct)**	ADHD	0.106	0.025	4.201	2.6×10^{-5}	0.057	0.156	**9.6×10^{-5}**
	Anxiety	0.012	0.025	0.473	0.636	–0.037	0.060	0.722
	MDD	0.010	0.025	0.358	0.720	–0.041	0.059	0.722
	CUD	0.055	0.025	2.191	0.003	0.006	0.105	0.062
	AUDIT-P	0.033	0.025	1.311	0.190	–0.016	0.083	0.308
	Smoking	0.117	0.026	4.507	6.6×10^{-6}	0.066	0.168	**4.3×10^{-5}**
	CognPerf	–0.015	0.025	–0.590	0.555	–0.063	0.034	0.721
	EA	–0.054	0.026	–2.060	0.039	–0.106	–0.003	0.073
	Income	–0.009	0.026	–0.356	0.722	–0.060	0.042	0.722
	AgeFirstBirth	–0.059	0.026	–2.258	0.024	–0.111	–0.008	0.062
	ASB	0.022	0.026	0.820	0.412	–0.030	0.073	0.595
	Risk	0.107	0.026	4.176	3.0×10^{-5}	0.057	0.157	**9.6×10^{-5}**
	Externalising	0.162	0.026	6.141	8.2×10^{-10}	0.110	0.214	**1.1×10^{-8}**
**Genetic transmission**	ADHD_f	0.051	0.012	4.196	2.7×10^{-5}	0.027	0.075	**1.0×10^{-4}**
	ADHD_m	0.051	0.012	4.197	2.7×10^{-5}	0.027	0.075	**1.0×10^{-4}**
	Anxiety_f	0.006	0.012	0.473	0.636	–0.018	0.030	0.722
	Anxiety_m	0.006	0.012	0.473	0.636	–0.018	0.029	0.722
	MDD_f	0.004	0.012	0.358	0.720	–0.019	0.028	0.722
	MDD_m	0.004	0.012	0.358	0.720	–0.020	0.029	0.722
	CUD_f	0.027	0.012	2.190	0.029	0.003	0.052	0.062
	CUD_m	0.026	0.012	2.189	0.029	0.003	0.050	0.062
	AUDIT-P_f	0.016	0.012	1.311	0.040	–0.008	0.040	0.309
	AUDIT-P_m	0.016	0.013	1.311	0.041	–0.008	0.041	0.309
	Smoking_f	0.057	0.013	4.494	7.0×10^{-6}	0.032	0.082	**4.5×10^{-5}**
	Smoking_m	0.058	0.013	4.494	7.0×10^{-6}	0.033	0.083	**4.5×10^{-5}**
	CognPerf_f	–0.007	0.013	–0.590	0.555	–0.031	0.017	0.721
	CognPerf_m	–0.007	0.013	–0.590	0.555	–0.030	0.016	0.721
	EA_f	–0.027	0.013	–2.059	0.040	–0.052	–0.001	0.073
	EA_m	–0.026	0.013	–2.059	0.040	–0.052	–0.001	0.073
	Income_f	–0.005	0.013	–0.356	0.722	–0.031	0.021	0.722
	Income_m	–0.004	0.013	–0.356	0.722	–0.029	0.020	0.722
	AgeFirstBirth_f	–0.029	0.013	–2.257	0.024	–0.054	–0.004	0.062
	AgeFirstBirth_m	–0.029	0.013	–2.257	0.024	–0.054	–0.004	0.062
	ASB_f	0.011	0.013	0.820	0.412	–0.015	0.036	0.595
	ASB_m	0.011	0.013	0.820	0.412	–0.015	0.036	0.595
	Risk_f	0.053	0.013	4.166	3.1×10^{-5}	0.028	0.078	**1.0×10^{-4}**
	Risk_m	0.052	0.013	4.165	3.1×10^{-5}	0.028	0.077	**1.0×10^{-4}**
	Externalising_f	0.078	0.013	6.108	1.0×10^{-9}	0.053	0.103	**1.3×10^{-8}**
	Externalising_m	0.079	0.013	6.108	1.0×10^{-9}	0.054	0.105	**1.3×10^{-8}**

Table S7 continued

Effect	PGS	*β*	*SE*	*Z*	*p*-value	CI lower	CI upper	*q*-value
**Genetic nurture**	ADHD_f	–0.010	0.022	–0.446	0.655	–0.053	0.034	0.838
	ADHD_m	0.015	0.021	0.714	0.475	–0.027	0.057	0.777
	Anxiety_f	0.054	0.022	2.436	0.015	0.011	0.098	0.386
	Anxiety_m	0.007	0.022	0.296	0.767	–0.037	0.050	0.889
	MDD_f	0.028	0.022	1.272	0.203	–0.015	0.071	0.707
	MDD_m	0.025	0.022	1.163	0.245	–0.017	0.067	0.707
	CUD_f	–0.015	0.022	–0.661	0.508	–0.058	0.029	0.777
	CUD_m	0.032	0.023	1.424	0.154	–0.012	0.077	0.707
	AUDIT-P_f	–0.005	0.021	–0.222	0.825	–0.046	0.037	0.889
	AUDIT-P_m	–0.004	0.022	–0.195	0.845	–0.048	0.040	0.889
	Smoking_f	0.010	0.023	–0.417	0.677	–0.036	0.056	0.838
	Smoking_m	0.012	0.022	0.532	0.595	–0.032	0.055	0.814
	CognPerf_f	–0.033	0.023	–1.445	0.148	–0.077	0.012	0.707
	CognPerf_m	0.015	0.022	0.700	0.484	–0.027	0.057	0.777
	EA_f	–0.012	0.023	–0.534	0.593	–0.057	0.032	0.814
	EA_m	0.018	0.023	0.768	0.442	–0.028	0.064	0.777
	Income_f	–0.018	0.022	–0.782	0.434	–0.061	0.026	0.777
	Income_m	–0.028	0.023	–1.221	0.222	–0.072	0.017	0.707
	AgeFirstBirth_f	–0.018	0.022	–0.843	0.399	–0.061	0.024	0.777
	AgeFirstBirth_m	0.004	0.022	0.183	0.855	–0.040	0.048	0.889
	ASB_f	0.022	0.023	0.979	0.328	–0.022	0.067	0.775
	ASB_m	0.028	0.023	1.213	0.225	–0.017	0.072	0.707
	Risk_f	0.024	0.023	1.055	0.291	–0.021	0.069	0.758
	Risk_m	0.041	0.024	1.725	0.084	–0.006	0.088	0.707
	Externalising_f	–0.002	0.022	–0.104	0.917	–0.046	0.041	0.917
	Externalising_m	0.033	0.023	1.463	0.144	–0.011	0.078	0.707

Note. Genetic transmission effects are equal for mothers and fathers. Genetic nurture effects: f = father, m = mother. *q*-values in bold indicate associations with $q < .05$ (FDR). CI lower = lower bound of the 95% confidence interval, CI upper = upper bound of the 95% confidence interval.

Table S8

Sensitivity Analysis Results for the Multi PGS Model Using Complete Data at Age 8 Years

Effect	PGS	*β*	*SE*	*Z*	*p*-value	CI lower	CI upper	*q*-value
**Child (direct)**	ADHD	0.121	0.016	7.804	6.0×10^{-15}	0.090	0.152	**3.6×10^{-14}**
	Anxiety	0.004	0.016	0.232	0.816	–0.027	0.034	0.816
	MDD	0.065	0.016	4.272	1.9×10^{-5}	0.034	0.095	**4.7×10^{-5}**
	CUD	0.045	0.016	2.814	0.005	0.013	0.077	**0.006**
	AUDIT-P	0.040	0.016	2.593	0.010	0.009	0.071	**0.010**
	Smoking	0.130	0.016	8.175	2.2×10^{-16}	0.098	0.161	**2.7×10^{-15}**
	CognPerf	–0.053	0.016	–3.443	0.001	–0.085	–0.022	**0.001**
	EA	–0.073	0.017	–4.513	6.4×10^{-6}	–0.106	–0.041	**1.9×10^{-5}**
	Income	–0.049	0.016	–3.161	0.002	–0.081	–0.018	**0.002**
	AgeFirstBirth	–0.058	0.017	–3.548	3.9×10^{-4}	–0.091	–0.025	**0.001**
	ASB	0.050	0.016	3.196	0.001	0.018	0.081	**0.002**
	Risk	0.100	0.016	6.512	7.4×10^{-11}	0.069	0.131	**3.0×10^{-10}**
**Genetic transmission**	ADHD_f	0.057	0.008	7.759	8.7×10^{-15}	0.043	0.072	**5.2×10^{-14}**
	ADHD_m	0.059	0.008	7.762	8.4×10^{-15}	0.044	0.074	**5.1×10^{-14}**
	Anxiety_f	0.002	0.008	0.232	0.816	–0.014	0.017	0.816
	Anxiety_m	0.002	0.008	0.232	0.816	–0.013	0.017	0.816
	MDD_f	0.031	0.008	4.264	2.0×10^{-5}	0.017	0.046	**4.8×10^{-5}**
	MDD_m	0.032	0.008	4.264	2.0×10^{-5}	0.017	0.047	**4.8×10^{-5}**
	CUD_f	0.022	0.008	2.812	0.005	0.006	0.038	**0.006**
	CUD_m	0.022	0.008	2.812	0.005	0.006	0.038	**0.006**
	AUDIT-P_f	0.020	0.008	2.591	0.010	0.004	0.035	**0.010**
	AUDIT-P_m	0.020	0.008	2.591	0.010	0.004	0.035	**0.010**
	Smoking_f	0.064	0.008	8.119	4.4×10^{-16}	0.048	0.080	**5.3×10^{-15}**
	Smoking_m	0.065	0.008	8.124	4.4×10^{-16}	0.049	0.082	**5.3×10^{-15}**
	CognPerf_f	–0.026	0.008	–3.439	0.001	–0.041	–0.011	**0.001**
	CognPerf_m	–0.026	0.008	–3.439	0.001	–0.041	–0.011	**0.001**
	EA_f	–0.037	0.008	–4.502	6.7×10^{-6}	–0.054	–0.021	**2.0×10^{-5}**
	EA_m	–0.035	0.008	–4.501	6.8×10^{-6}	–0.051	–0.020	**2.0×10^{-5}**
	Income_f	–0.025	0.008	–3.158	0.002	–0.041	–0.009	**0.002**
	Income_m	–0.025	0.008	–3.158	0.002	–0.040	–0.009	**0.002**
	AgeFirstBirth_f	–0.029	0.008	–3.544	3.9×10^{-4}	–0.045	–0.012	**0.001**
	AgeFirstBirth_m	–0.029	0.008	–3.544	3.9×10^{-4}	–0.045	–0.013	**0.001**
	ASB_f	0.024	0.008	3.194	0.001	0.009	0.040	**0.002**
	ASB_m	0.025	0.008	3.194	0.001	0.009	0.041	**0.002**
	Risk_f	0.049	0.008	6.485	8.9×10^{-11}	0.034	0.064	**3.6×10^{-10}**
	Risk_m	0.050	0.008	6.486	8.8×10^{-11}	0.035	0.066	**3.5×10^{-10}**

Table S8 continued

Effect	PGS	β	*SE*	*Z*	*p*-value	CI lower	CI upper	*q*-value
**Genetic nurture**	ADHD_f	–0.0002	0.015	–0.013	0.989	–0.030	0.029	1.000
	ADHD_m	0.016	0.015	1.108	0.268	–0.013	0.046	0.413
	Anxiety_f	0.005	0.015	0.378	0.706	–0.024	0.035	0.891
	Anxiety_m	–0.007	0.015	–0.512	0.609	–0.037	0.022	0.812
	MDD_f	–0.048	0.015	–3.221	0.001	–0.078	–0.018	**0.006**
	MDD_m	–0.021	0.015	–1.424	0.154	–0.052	0.009	0.285
	CUD_f	–0.024	0.014	–1.713	0.087	–0.052	0.004	0.189
	CUD_m	–0.028	0.015	–1.942	0.052	–0.056	0.001	0.139
	AUDIT-P_f	3.0×10^{-5}	0.015	2.0×10^{-4}	1.000	–0.028	0.028	1.000
	AUDIT-P_m	–0.005	0.014	–0.325	0.745	–0.033	0.024	0.894
	Smoking_f	–0.054	0.016	–3.365	0.001	–0.086	–0.022	**0.005**
	Smoking_m	–0.030	0.016	–1.862	0.063	–0.062	0.002	0.150
	CognPerf_f	0.003	0.016	0.216	0.829	–0.028	0.034	0.948
	CognPerf_m	0.054	0.016	3.528	4.2×10^{-4}	0.023	0.085	**0.003**
	EA_f	0.039	0.018	2.270	0.023	0.004	0.074	0.070
	EA_m	0.076	0.018	4.282	1.9×10^{-5}	0.040	0.112	**4.4×10^{-4}**
	Income_f	0.018	0.016	1.142	0.253	–0.013	0.049	0.413
	Income_m	–0.001	0.015	–0.074	0.941	–0.031	0.029	1.000
	AgeFirstBirth_f	0.023	0.016	1.480	0.139	–0.008	0.055	0.278
	AgeFirstBirth_m	0.037	0.016	2.321	0.020	0.005	0.069	0.070
	ASB_f	–0.015	0.015	–1.091	0.275	–0.044	0.013	0.413
	ASB_m	–0.011	0.015	–0.765	0.445	–0.040	0.018	0.628
	Risk_f	–0.036	0.015	–2.420	0.016	–0.067	–0.006	0.062
	Risk_m	–0.060	0.016	–3.855	0.000	–0.091	–0.029	**0.001**

Note. Genetic transmission effects are equal for mothers and fathers. Genetic nurture effects: f = father, m = mother. *q*-values in bold indicate associations with $q < .05$ (FDR). CI lower = lower bound of the 95% confidence interval, CI upper = upper bound of the 95% confidence interval.

Table S9

Correlations Between Child, Maternal and Paternal Polygenic Scores

PGS	Mother ~~ Father	Mother ~~ Child	Father ~~ Child
ADHD	0.03	0.51	0.50
Anxiety	–0.01	0.49	0.49
Depression	<0.01	0.49	0.49
Cannabis use disorder	<0.01	0.49	0.50
Problematic alcohol use	–0.01	0.49	0.49
Smoking	0.04	0.52	0.51
Cognitive performance	0.04	0.51	0.51
Educational attainment	0.14	0.55	0.56
Income	0.04	0.52	0.51
Age at first birth	0.07	0.52	0.53
Antisocial behaviour	–0.02	0.49	0.49
Risk-taking	0.02	0.50	0.50
Externalising	0.05	0.52	0.51

Note. Correlations between maternal and paternal polygenic scores are expected to be close to 0 (under random mating). Non-zero correlations likely indicate assortative mating or population stratification (e.g., for educational attainment). Correlations between parental polygenic scores and child polygenic scores are expected to be close to 0.5. Correlations higher/lower than 0.5 reflect the positive/negative correlations between parental polygenic scores, with the largest deviation also found for polygenic scores for educational attainment.

References

1. Delaneau O, Marchini J, Zagury JF. A linear complexity phasing method for thousands of genomes. Nat Methods. 2012 Feb;9(2):179–81.

2. Marchini Group. IMPUTE4 [Internet]. Available from: https://jmarchini.org/software/#impute-4.

3. McCarthy S, Das S, Kretzschmar W, Delaneau O, Wood AR, Teumer A, et al. A reference panel of 64,976 haplotypes for genotype imputation. Nat Genet. 2016 Oct;48(10):1279–83.

4. Woods AD, Gerasimova D, Van Dusen B, Nissen J, Bainter S, Uzdavines A, et al. Best practices for addressing missing data through multiple imputation. Infant Child Dev. 2023;n/a(n/a):e2407.

5. Achenbach TM. Manual for the Child Behavior Checklist/2-3 and 1992 profile. Burlingt VT Univ Vt Dep Psychiatry. 1992;

6. Eilertsen EM, Cheesman R, Ayorech Z, Røysamb E, Pingault JB, Njølstad PR, et al. On the importance of parenting in externalizing disorders: an evaluation of indirect genetic effects in families. J Child Psychol Psychiatry. 2022;63(10):1186–95.

7. Bijma P. The quantitative genetics of indirect genetic effects: a selective review of modelling issues. Heredity. 2014 Jan;112(1):61–9.

8. Cheesman R, Selzam S, Ronald A, Dale PS, McAdams TA, Eley TC, et al. Childhood behaviour problems show the greatest gap between DNA-based and twin heritability. Transl Psychiatry. 2017 Dec 12;7(12):1–9.

9. Kong A, Thorleifsson G, Frigge ML, Vilhjalmsson BJ, Young AI, Thorgeirsson TE, et al. The nature of nurture: Effects of parental genotypes. Science. 2018;359(6374):424–8.

10. Wang B, Baldwin JR, Schoeler T, Cheesman R, Barkhuizen W, Dudbridge F, et al. Robust genetic nurture effects on education: A systematic review and meta-analysis based on 38,654 families across 8 cohorts. Am J Hum Genet. 2021 Sep 2;108(9):1780–91.

11. Tubbs JD, Zhang YD, Sham PC. Intermediate confounding in trio relationships: The importance of complete data in effect size estimation. Genet Epidemiol. 2020;44(4):395–9.

12. Demontis D, Walters RK, Martin J, Mattheisen M, Als TD, Agerbo E, et al. Discovery of the first genome-wide significant risk loci for attention deficit/hyperactivity disorder. Nat Genet. 2019 Jan;51(1):63–75.

13. Purves KL, Coleman JRI, Meier SM, Rayner C, Davis KAS, Cheesman R, et al. A major role for common genetic variation in anxiety disorders. Mol Psychiatry [Internet]. 2019 Nov 20 [cited 2020 Mar 10]; Available from: http://www.nature.com/articles/s41380-019-0559-1

14. Howard DM, Adams MJ, Clarke TK, Hafferty JD, Gibson J, Shirali M, et al. Genome-wide meta-analysis of depression identifies 102 independent variants and highlights the importance of the prefrontal brain regions. Nat Neurosci. 2019 Mar;22(3):343–52.

15. Lee JJ, Wedow R, Okbay A, Kong E, Maghzian O, Zacher M, et al. Gene discovery and polygenic prediction from a genome-wide association study of educational attainment in 1.1 million individuals. Nat Genet. 2018 Aug;50(8):1112–21.

16. Watanabe K, Stringer S, Frei O, Umićević Mirkov M, de Leeuw C, Polderman TJC, et al. A global overview of pleiotropy and genetic architecture in complex traits. Nat Genet. 2019 Sep;51(9):1339–48.

17. Johnson EC, Demontis D, Thorgeirsson TE, Walters RK, Polimanti R, Hatoum AS, et al. A large-scale genome-wide association study meta-analysis of cannabis use disorder. Lancet Psychiatry. 2020 Dec 1;7(12):1032–45.

18. Sanchez-Roige S, Palmer AA, Fontanillas P, Elson SL, Adams MJ, Howard DM, et al. Genome-Wide Association Study Meta-Analysis of the Alcohol Use Disorders Identification Test (AUDIT) in Two Population-Based Cohorts. Am J Psychiatry. 2019 Feb 1;176(2):107–18.

19. Mills MC, Tropf FC, Brazel DM, van Zuydam N, Vaez A, Pers TH, et al. Identification of 371 genetic variants for age at first sex and birth linked to externalising behaviour. Nat Hum Behav. 2021 Jul 1;1–14.

20. Tielbeek JJ, Uffelmann E, Williams BS, Colodro-Conde L, Gagnon É, Mallard TT, et al. Uncovering the genetic architecture of broad antisocial behavior through a genome-wide association study meta-analysis. Mol Psychiatry. 2022 Oct 25;1–11.

21. Linnér RK, Biroli P, Kong E, Meddens SFW, Wedow R, Fontana MA, et al. Genome-wide association analyses of risk tolerance and risky behaviors in over 1 million individuals identify hundreds of loci and shared genetic influences. Nat Genet. 2019 Feb;51(2):245–57.

22. Bulik-Sullivan BK, Loh PR, Finucane HK, Ripke S, Yang J, Patterson N, et al. LD Score regression distinguishes confounding from polygenicity in genome-wide association studies. Nat Genet. 2015 Mar;47(3):291–5.

23. Linnér RK, Mallard TT, Barr PB, Sanchez-Roige S, Madole JW, Driver MN, et al. Multivariate analysis of 1.5 million people identifies genetic associations with traits related to self-regulation and addiction. Nat Neurosci. 2021 Oct;24(10):1367–76.

24. Williams CM, Poore H, Tanksley PT, Kweon H, Courchesne-Krak NS, Londono-Correa D, et al. Guidelines for Evaluating the Comparability of Down-Sampled GWAS Summary Statistics. Behav Genet. 2023 Nov 1;53(5):404–15.
